# Effects of FSGS-associated mutations on the stability and function of myosin-1 in fission yeast

**DOI:** 10.1242/dmm.020214

**Published:** 2015-08-01

**Authors:** Jing Bi, Robert T. Carroll, Michael L. James, Jessica L. Ouderkirk, Mira Krendel, Vladimir Sirotkin

**Affiliations:** Department of Cell and Developmental Biology, SUNY Upstate Medical University, 750 E. Adams St., Syracuse, NY 13210, USA

**Keywords:** FSGS, Myosin, *S. pombe*, Eisosomes

## Abstract

Point mutations in the human *MYO1E* gene, encoding class I myosin Myo1e, are associated with focal segmental glomerulosclerosis (FSGS), a primary kidney disorder that leads to end-stage kidney disease. In this study, we used a simple model organism, fission yeast *Schizosaccharomyces pombe*, to test the effects of FSGS-associated mutations on myosin activity. Fission yeast has only one class I myosin, Myo1, which is involved in actin patch assembly at the sites of endocytosis. The amino acid residues mutated in individuals with FSGS are conserved between human Myo1e and yeast Myo1, which allowed us to introduce equivalent mutations into yeast myosin and use the resulting mutant strains for functional analysis. Yeast strains expressing mutant Myo1 exhibited defects in growth and endocytosis similar to those observed in the *myo1* deletion strain. These mutations also disrupted Myo1 localization to endocytic actin patches and resulted in mis-localization of Myo1 to eisosomes, linear membrane microdomains found in yeast cells. Although both mutants examined in this study exhibited loss of function, one of these mutants was also characterized by the decreased protein stability. Thus, using the yeast model system, we were able to determine that the kidney-disease-associated mutations impair myosin functional activity and have differential effects on protein stability.

## Abstract (precis)

**Summary:** In the fission yeast *S. pombe*, kidney disease-associated mutations in Myo1, a homolog of human Myo1e, disrupt myosin localization and function.

## INTRODUCTION

Missense mutations in the human *MYO1E* gene, encoding molecular motor protein myosin 1e (Myo1e), are associated with kidney disease (Al-Hamed et al., 2013; Mele et al., 2011; Sanna-Cherchi et al., 2011). Although these mutations have been predicted to disrupt Myo1e activity, testing this prediction directly in a vertebrate model organism is challenging, not only owing to the need for complex genetic modifications but also because of the limitations of microscopic and biochemical characterization in such a model. Therefore, we took advantage of a favorable model system, fission yeast *Schizosaccharomyces* *pombe*, to examine the effects of disease-associated mutations on myosin-1 localization, activity and stability.

From more than 20 known classes of myosins, approximately 40 myosins distributed among 12 classes are present in humans (Berg et al., 2001), including eight members of class I myosins. Several myosins have been implicated in human health and disease (Moore et al., 2012; Ouderkirk and Krendel, 2014; Redowicz, 2002; Tajsharghi and Oldfors, 2013). Mutations in the *MYO1E* gene in humans are associated with focal segmental glomerulosclerosis (FSGS) (Al-Hamed et al., 2013; Mele et al., 2011; Sanna-Cherchi et al., 2011), a kidney disease arising from the disruption of the protein filtration barrier in the kidney, which leads to proteinuria (protein excretion in the urine). FSGS is characterized by the structural changes in the renal glomeruli, the portions of the nephrons that are responsible for selective protein filtration. In many cases of familial FSGS, the disruption of renal filtration arises from the defects in specialized epithelial cells of the glomerulus, called podocytes (Greka and Mundel, 2012). Podocytes have long, actin-rich, interdigitating cell processes, called foot processes, which cover the entire surface of the glomerular capillaries. The contacts between podocyte foot processes, also known as the slit diaphragms, represent one of the key components of the glomerular filtration barrier (George and Holzman, 2012; Greka and Mundel, 2012).

Myo1e is expressed in podocytes and plays a key role in regulating the integrity of their cell-cell junctions (Bi et al., 2013). *Myo1e*-null mice develop proteinuria at 2 weeks after birth, along with the structural changes in the glomeruli that are reminiscent of those observed in individuals with FSGS (Krendel et al., 2009; Mele et al., 2011). Although it is known that the complete knockout of *Myo1e* or its selective knockout in podocytes are sufficient to induce FSGS-like disease in mice (Chase et al., 2012; Krendel et al., 2009), the effects of substituting wild-type *Myo1e* with the mutant versions associated with human FSGS have not been tested in a model organism.

**Fig. 1.**
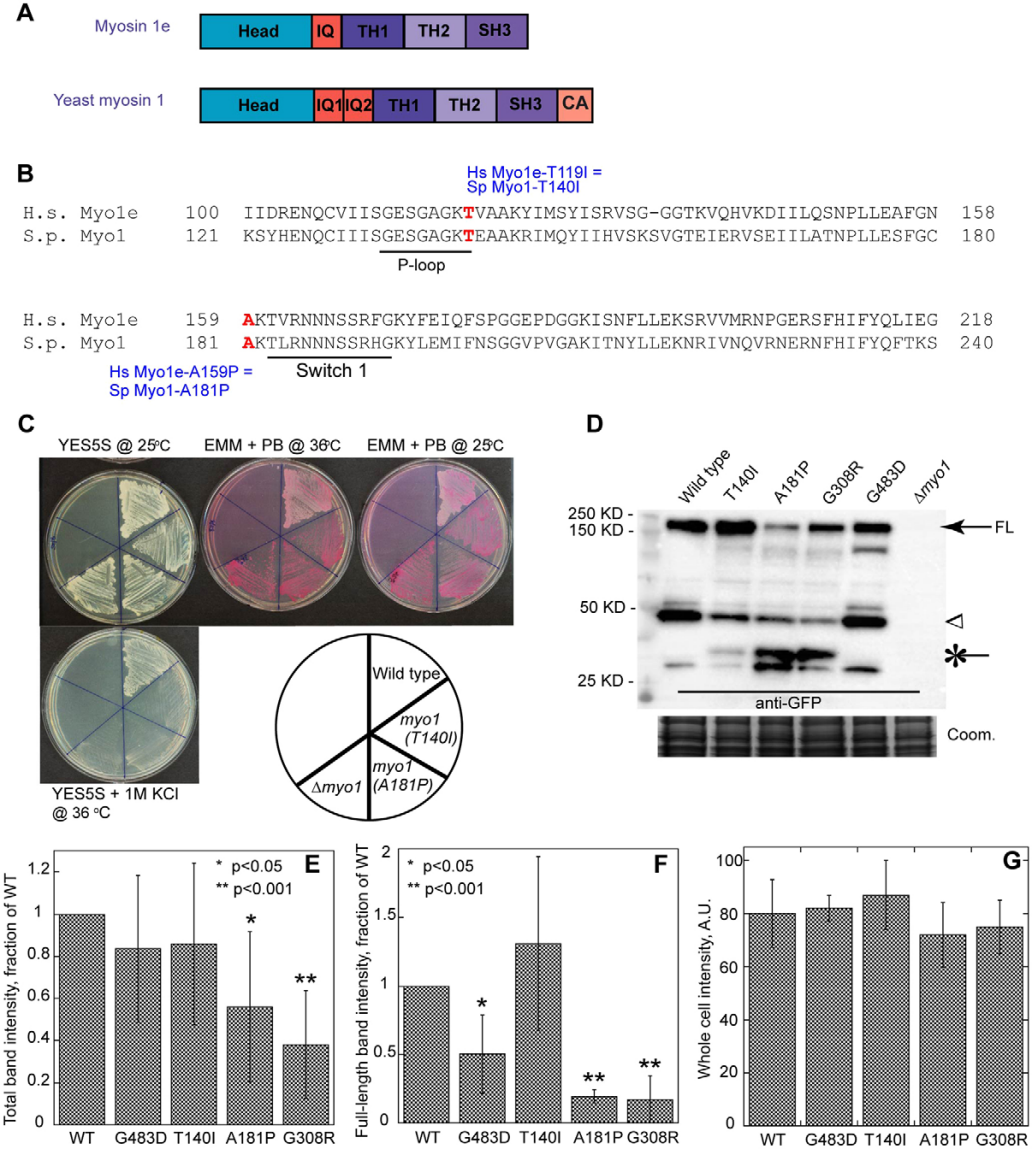
**Kidney-disease-associated mutations in *S. pombe* Myo1 result in growth defects and decreased protein stability.** (A) Domain maps of *H. sapiens* Myo1e and *S. pombe* Myo1. IQ, light-chain-binding IQ motif; TH, tail homology domain; SH3, Src homology 3 domain; CA, central-acidic domain. (B) Alignment of amino acid sequences of the N-terminal portions of *H. sapiens* (*H.s.*) myosin 1e (Myo1e) and *S. pombe* (*S.p.*) myosin-1 (Myo1). Red bold font indicates the sites of the two FSGS-associated mutations. The conserved P-loop motif and Switch I motifs are underlined. The T119I and A159P mutations in *H.s.* Myo1e are equivalent to the T140I and A181P mutations in *S.p.* Myo1, respectively. (C) Analysis of the salt and temperature sensitivity of the wild-type and untagged *myo1* mutant strains. The *myo1(T140I)*, *myo1(A181P)* and *Δmyo1* strains fail to grow in the presence of high salt at high temperature (YE5S+1 M KCl at 36°C) and grow but stain darkly with Phloxine B (PB) on minimal EMM medium at 25°C and 36°C, indicating decreased cell viability. (D-G) Western blot analysis of yeast extracts using anti-GFP antibody to determine protein stability of the mGFP-tagged Myo1 constructs. (D) Similar amounts of the full-length Myo1-mGFP (FL, arrow) are detected in cells expressing mGFP-tagged wild-type, T140I or G483D mutant Myo1, whereas the amount of the full-length myosin is reduced in the strains expressing the A181P and G308R Myo1 mutants. An arrowhead indicates the ∼40-kDa degradation product. An asterisk indicates a lower ∼30-kDa molecular weight degradation product that is enriched in the A181P and G308R mutants. No signal is detected in the *Δmyo1* strain. Coomassie-stained gel (bottom panel) served as the loading control. (E,F) Quantification of (E) total band intensity and (F) full-length band intensity normalized to the wild type. *n*=5 blots from 3 independent experiments. Error bars represent s.d. (G) The background-subtracted whole-cell intensity of mGFP-tagged Myo1 variants in cells before lysis. *n*=5 cells. Error bars represent s.d. *P<0.05; **P<0.001.

Like other members of the myosin superfamily, human Myo1e and *S. pombe* class I myosin Myo1 have a highly conserved N-terminal motor domain (Fig. 1A) that uses ATP hydrolysis to move along an actin filament and a C-terminal tail domain that binds membrane and protein cargo (Bement et al., 1994; El Mezgueldi et al., 2002; Feeser et al., 2010; Krendel et al., 2007; Lee et al., 2000; Sirotkin et al., 2005). Both missense mutations in the *MYO1E* gene identified in individuals with FSGS result in changes in conserved amino acid residues in the myosin motor domain. The T119I mutation (Al-Hamed et al., 2013) is located in the P-loop region, whereas the A159P mutation (Mele et al., 2011; Sanna-Cherchi et al., 2011) is located adjacent to the Switch-I region (Fig. 1B). These regions are involved in ATP binding and hydrolysis. Therefore, mutations in these highly conserved residues are likely to disrupt the functions of the myosin motor domain.

In order to determine whether a particular disease-associated mutation is pathogenic or represents a non-pathogenic polymorphism, it is important to test the effects of each mutation on protein functions. Although the effects of T119I on myosin-1 function are yet to be tested, we have previously characterized the effects of the A159P mutation on Myo1e activity by expressing the mutant Myo1e in the *Myo1e*-null podocytes (Bi et al., 2013). The mutant was mis-localized, and cells expressing this Myo1e variant exhibited defects in cell-cell contact formation. However, this analysis was complicated by the inherent variability of contact assembly between individual pairs of cells detected by live cell imaging and by the lack of a clear understanding of Myo1e function in contact assembly.

**TRANSLATIONAL IMPACT**

**Clinical issue**

Mutations in the human *MYO1E* gene are associated with focal segmental glomerulosclerosis (FSGS), a chronic kidney disease characterized by defects in glomerular filtration. The *MYO1E* gene encodes molecular motor myosin 1e, which is expressed in glomerular epithelial cells. In order to assess the pathogenicity of specific *MYO1E* mutations, it is important to determine the effects of these mutations on myosin 1e activity, a task that can be challenging to perform in animal models or cell cultures. Because the amino acid residues that are affected by the mutations found in individuals with FSGS are conserved between human myosin 1e and fission yeast myosin-1 (Myo1), yeast can be used as a model system to determine the effects of FSGS-associated mutations on myosin-1 activity.

**Results**

Two point mutations equivalent to those found in individuals with FSGS were individually introduced into yeast *myo1*. Wild-type Myo1 is involved in endocytosis and localizes to actin patches, the sites of endocytosis in fission yeast. Both mutant strains exhibited defects in growth and endocytosis similar to those observed in the yeast strain lacking Myo1. Myo1 mutants did not localize to endocytic actin patches. Whereas both mutations analyzed in this study disrupted Myo1 localization and function, only one also decreased the protein stability.

**Implications and future directions**

This study shows that FSGS-associated mutations in the Myo1 motor domain disrupt Myo1 activity and intracellular localization. This suggests that myosin-1 motor activity might play an important role in maintaining normal glomerular filtration. In order to better understand the pathogenesis of FSGS and guide the identification of potential therapy targets for FSGS, it will be important to determine how myosin 1e motor activity contributes to the function of the glomerular filtration barrier.

In the present study, we tested the effects of mutations equivalent to a known Myo1e pathogenic mutation (A159P) and a novel mutation recently identified by candidate gene sequencing (T119I) (Al-Hamed et al., 2013) in a favorable model system, *S. pombe*. Unlike vertebrates, which have multiple class I myosins, fission yeast have a single class 1 myosin, Myo1, and its localization and function are well-defined. Myo1 assembles into actin patches at the sites of endocytosis in an ordered, highly reproducible manner and plays a role in promoting endocytic internalization (Sirotkin et al., 2010). Yeast lacking Myo1 exhibit growth defects, abnormal morphology, and changes in actin patch localization and dynamics (Lee et al., 2000; Sirotkin et al., 2005). We took advantage of the conservation of motor domains between Myo1e and Myo1 to examine the effects of the FSGS-equivalent mutations on fission yeast Myo1. Intriguingly, we found that, although both mutations disrupted Myo1 localization and function, they had differential effects on Myo1 stability and folding as gauged by immunoblotting and by colocalization with a myosin chaperone, Rng3 (Lord and Pollard, 2004; Stark et al., 2013).

## RESULTS

### Mutations in the motor domain of Myo1, equivalent to the FSGS-associated Myo1e mutations, result in yeast growth defects

Based on sequence alignment (Fig. 1B), we identified conserved residues T140 and A181 in the *S. pombe* Myo1 motor domain as homologous to the residues T119 and A159 in human Myo1e, which are disrupted by the T119I and A159P mutations in humans with FSGS. To examine the effects of these mutations in fission yeast, we replaced the wild-type *myo1^+^* coding sequence with the mutant *myo1* variants directly in the yeast genome so that the mutant myosins were expressed from the *myo1* genomic locus under the control of the endogenous promoter. Like *Δmyo1* (Lee et al., 2000), *myo1(T140I)* and *myo1(A181P)* cells were viable and were able to grow on rich YES medium at 25°C (Fig. 1C).

Many endocytosis mutants in budding and fission yeast, including *Δmyo1* (Lee et al., 2000; Munn et al., 1995; Whitacre et al., 2001), exhibit sensitivity to high temperature (36°C) and high salt (1 M KCl or NaCl). To examine the effects of Myo1 motor domain mutations on cell growth, we directly compared the ability of *myo1(T140I)*, *myo1(A181P)*, *Δmyo1* and wild-type cells to grow on rich YES medium containing 1 M KCl at 36°C and on minimal EMM medium containing Phloxine B (PB), an indicator of cell viability, at 25°C and 36°C (Fig. 1C). Increased PB staining indicates decreased viability. Wild-type cells grew on YES+1 M KCl plates and stained lightly pink on EMM+PB plates at 25°C and 36°C. In contrast, *myo1(T140I)* and *myo1(A181P)* cells completely failed to grow on YES+1 M KCl plates and grew but stained dark red on EMM+PB plates at both 25°C and 36°C, indicating decreased viability. Salt sensitivity and decreased viability of *myo1(T140I)* and *myo1(A181P)* cells were identical to those observed in *Δmyo1* cells. Thus, each of the two motor domain mutations disrupted Myo1 function in yeast cells to the same degree as complete *myo1* deletion.

### A181P but not T140I mutation results in decreased stability of Myo1 in yeast

To examine the effects of motor domain mutations on Myo1 stability and localization in cells, we tagged both mutants with monomeric GFP (mGFP) or mCherry at the C-terminus directly in the native *myo1* chromosomal location in haploid cells. The tagged proteins were expressed under the control of the endogenous *myo1* promoter and were the sole source of Myo1 in cells. We compared the stability and localization of mGFP-tagged proteins to similarly tagged wild-type Myo1 and two previously constructed Myo1 motor domain mutants, Myo1-E1 (G308R) and Myo1-S1 (G483D) (Stark et al., 2013). The Myo1-E1 and Myo1-S1 mutants were modeled after the severe Myo2-E1 and mild Myo2-S1 mutants of *S. pombe* myosin-II Myo2 (Balasubramanian et al., 1998; Wong et al., 2000). The G345R mutation in the *myo2-E1* allele destabilizes Myo2, resulting in cytokinesis defects and enhanced recruitment of the myosin chaperone Rng3 to the contractile ring, whereas the G515D mutation in the *myo2-S1* allele does not (Stark et al., 2013; Wong et al., 2000).

Western blot analysis of yeast extracts using anti-GFP antibodies revealed increased degradation of the mGFP-tagged A181P and G308R Myo1 mutants compared to the mGFP-tagged wild-type Myo1, and T140I and G483D Myo1 mutants (Fig. 1D-F and supplementary material Fig. S1A). Under our lysis conditions, even wild-type Myo1-mGFP was susceptible to degradation such that 30% was detected as the ∼150-kDa full-length Myo1-mGFP (arrow in Fig. 1D and supplementary material Fig. S1A), and the rest as lower molecular weight degradation products with a prominent ∼40 kDa band (arrowhead in Fig. 1D). A similar degradation pattern and similar amounts of the total mGFP-tagged protein were observed for Myo1(T140I)-mGFP and Myo1(G483D)-mGFP, although, compared to the wild-type Myo1-mGFP, more Myo1(T140I)-mGFP and less Myo1(G483D)-mGFP were detected as the full-length protein (Fig. 1D and supplementary material Fig. S1A). In contrast, the total amounts of Myo1(A181P)-mGFP and Myo1(G308R)-mGFP were substantially reduced to 0.6 and 0.4 of the wild-type level (Fig. 1E), respectively, and only 10% of the total was detected as the full-length protein (Fig. 1F and supplementary material Fig. S1A). Myo1(A181P)-mGFP and Myo1(G308R)-mGFP also showed a different degradation pattern: the amounts of the 40-kDa degradation product were greatly reduced and replaced with a new, lower molecular weight ∼30-kDa product (asterisk in Fig. 1D), representing almost complete degradation of mutant Myo1. Unlike the level of protein detected by immunoblotting, the total fluorescence intensities of cells before lysis (Fig. 1G), representing total intracellular concentrations (Wu and Pollard, 2005), were the same for all strains, indicating that all mutants were expressed at the same level, and that the degradation likely occurred in part after cell lysis. Treatment with 0.1 mg/ml cycloheximide for 24 h to block new protein synthesis had no effect on the degradation pattern and amounts of the detected protein (supplementary material Fig. S1B), suggesting that, in cells, the turnover of wild-type and mutant Myo1 is very slow and their degradation products, once generated, remain stable in the cytoplasm. Increased degradation detected by immunoblotting suggested decreased stability of A181P and G308R Myo1 mutants. Surprisingly, from the two FSGS-associated mutations, only the A181P mutation decreased Myo1 stability, whereas the T140I mutation did not.

### Myo1 motor domain mutations disrupt localization and function of Myo1 in endocytic actin patches

**Fig. 2.**
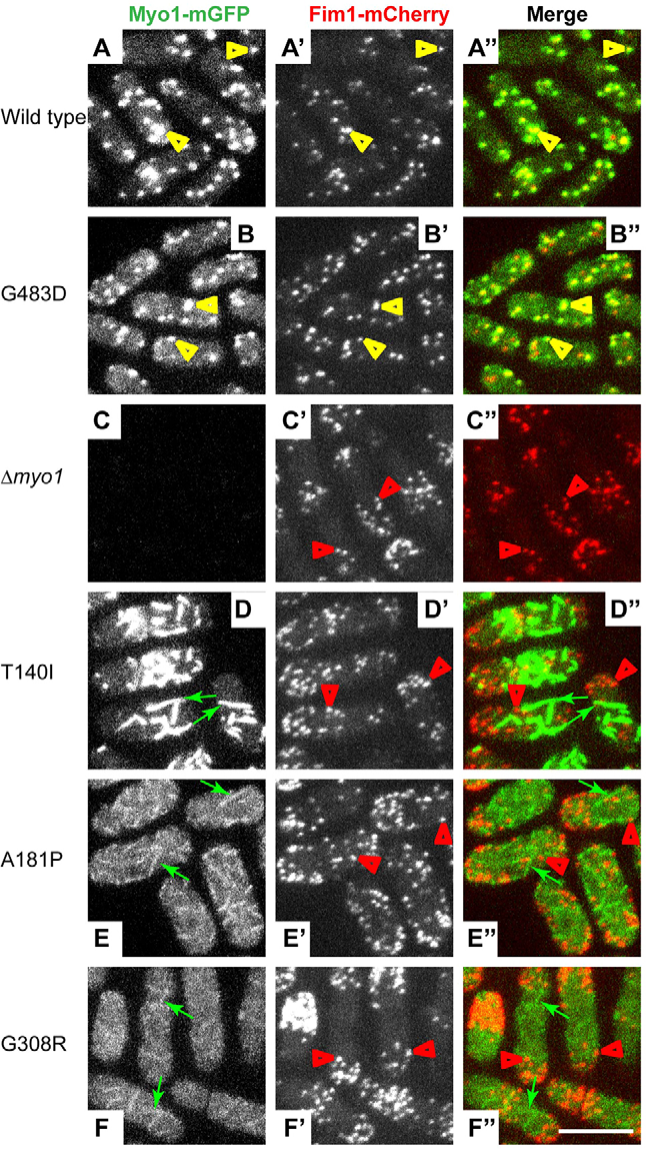
**Motor domain mutations disrupt Myo1 localization to actin patches.** Confocal images show the localization of (left panels, A-F) mGFP-tagged (A) wild-type or (B-F) mutant Myo1, (middle panels, A′-F′) mCherry-tagged fimbrin (Fim1-mCherry), and (right panels, A″-F″) merge of Myo1-mGFP (green) and Fim1-mCherry (red) images. Wild-type Myo1 and Myo1(G483D) colocalize with Fim1-mCherry in actin patches (yellow arrowheads), whereas Myo1(T140I), Myo1(A181P) and Myo1(G308R) localize to eisosomes (green arrows) and are absent from actin patches (red arrowheads). The images represent maximum intensity projections of three consecutive optical sections through the top surface of the cell acquired at 0.4-µm intervals. Scale bar: 10 µm.

To characterize the effects of motor domain mutations on Myo1 localization in live fission yeast cells, we examined localization of mGFP-tagged wild-type and mutant Myo1 variants relative to the endocytic actin patches labeled with mCherry-tagged actin-binding protein fimbrin (Fim1; Fig. 2). Whereas the wild-type Myo1-mGFP and the mild mutant Myo1(G483D)-mGFP colocalized with Fim1-mCherry in more than 95% endocytic actin patches on the cell surface (supplementary material Fig. S2A and yellow arrowheads in Fig. 2A-B″), the three other motor domain mutants, Myo1(T140I)-mGFP, Myo1(A181P)-mGFP and Myo1(G308R)-mGFP, did not (Fig. 2D-F″). Instead, these three Myo1 motor domain mutants localized to filamentous structures at the cell cortex (green arrows in Fig. 2D-F″), which had only nominal, less than 10%, overlap with actin patches (supplementary material Fig. S2A). Thus, a functional motor domain is required for Myo1 localization to actin patches. Interestingly, approximately threefold more Myo1(T140I)-mGFP localized to these filamentous cortical structures, subsequently identified as eisosomes, than Myo1(A181P)-mGFP or Myo1(G308R)-mGFP (supplementary material Fig. S2B-E), which correlated with the amount of full-length protein detected by immunoblotting (Fig. 1 and supplementary material Fig. S1). This suggests that the increased degradation of A181P and G308R Myo1 mutants decreases their localization at the cell cortex.

**Fig. 3.**
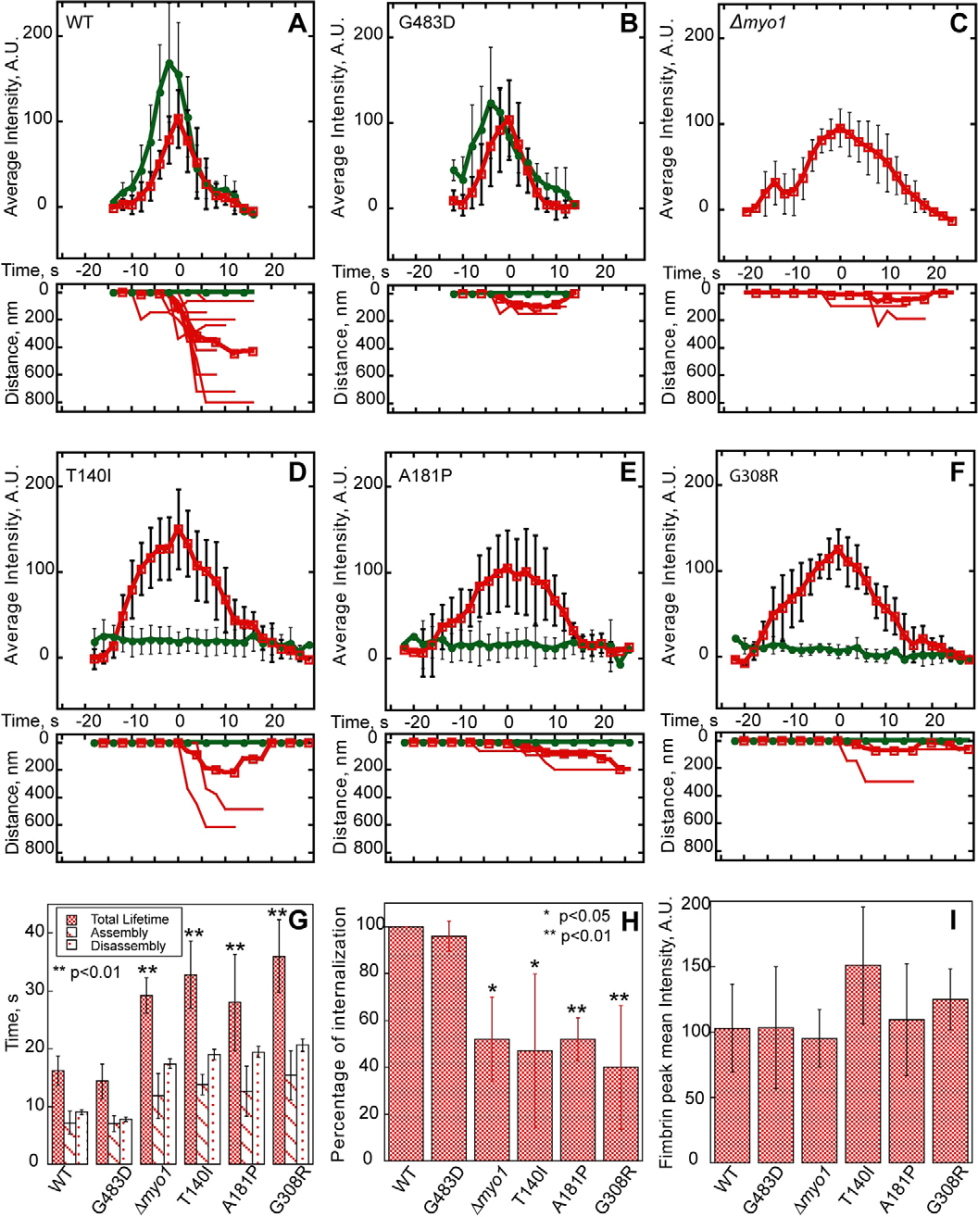
**Motor domain mutations disrupt dynamics of endocytic actin patches visualized with mCherry-tagged fimbrin (Fim1).** (A-F) Time courses of (upper panels) average fluorescence intensity of (green, solid circles) wild-type (WT) and mutant Myo1-mGFP and (red, open squares) Fim1-mCherry in actin patches, and (lower panels) raw (thin lines) and average (thick lines) distances traveled by Fim1-mCherry or Myo1-mGFP in patches. (G-I) Bar graphs showing (G) the total lifetime, assembly time and disassembly time, (H) percentage of internalization, and (I) mean peak intensities of Fim1-mCherry patches in wild-type and mutant *myo1* strains. Note increased total lifetime, assembly and disassembly times, and decreased internalization of Fim1-mCherry patches in the T140I, A181P and G308R *myo1* mutant, and *Δmyo1* strains. In all panels, error bars represent s.d. (A-G,I) n=5-12 patches. (H) n=23-43 patches in 3-6 cells. The asterisks indicate statistical significance: *P<0.05; **P<0.01.

Because Myo1(T140I)-mGFP, Myo1(A181P)-mGFP and Myo1(G308R)-mGFP failed to localize to actin patches, we compared actin patch dynamics measured with Fim1-mCherry in Myo1 motor domain mutants, *Δmyo1* or wild-type cells (Fig. 3A-F). In the wild-type cells, patches assembled in ∼7 s to peak intensity corresponding to ∼900 fimbrin molecules (Sirotkin et al., 2010) and disassembled in 9 s, and nearly all patches moved away from the cortex, representing successful endocytic internalization. The wild-type Myo1 appeared 1-2 s before Fim1, peaked 1-2 s before Fim1, and stayed on the membrane as actin patch associated with the endocytic vesicle internalized (Fig. 3A), which is why colocalization between Myo1 and Fim1 is not perfect (Sirotkin et al., 2005). The same actin patch dynamics were observed in cells expressing the mild Myo1(G483D)-mGFP mutant (Fig. 3B). In contrast, mGFP-tagged T140I, A181P and G308R Myo1 mutants completely failed to localize to actin patches at any point during the patch lifetime (Fig. 3D-F), and cells expressing these mutants exhibited the same defects in patch assembly and constitutive endocytic internalization (Fig. 3G-I) as in the *Δmyo1* cells (Fig. 3C). In cells expressing Myo1(T140I)-mGFP, Myo1(A181P)-mGFP or Myo1(G308R)-mGFP, patches exhibited a 12-20 s longer lifetime (T140I: 32.8±5.8 s; A181P: 28.0±8.4 s; G308R: 36.0±6.3 s) due to a 6-8 s longer assembly time (T140I: 13.8±1.8 s; A181P: 12.6±4.3 s; G308R: 15.4±4.3 s) and 6-12 s longer disassembly time compared to the wild-type cells (Fig. 3G). Although patches in these three motor domain mutants assembled to the similar peak intensity as in the wild-type cells (Fig. 3I), only half of the patches successfully internalized (Fig. 3H), indicating a significant defect in constitutive endocytosis, which correlated with the reduced viability of these cells (Fig. 1C). Thus, motor domain mutations prevent Myo1 localization to patches and result in the same defects of actin patch dynamics as the complete *myo1* deletion.

### Myo1 motor domain mutants colocalize with the eisosome marker Fhn1

**Fig. 4.**
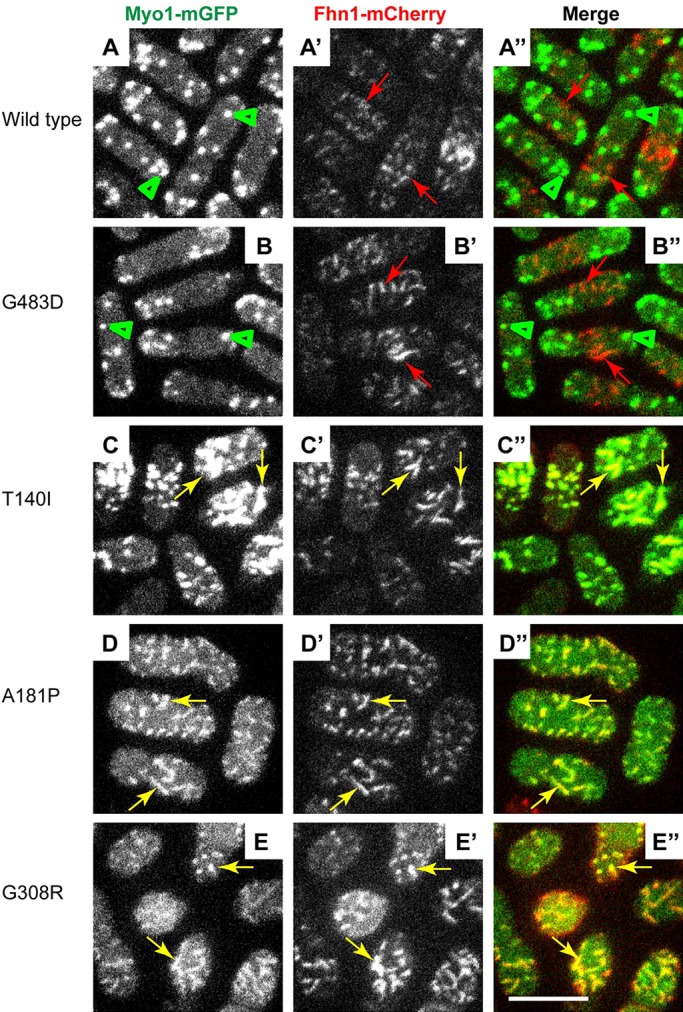
**Myo1 motor domain mutants colocalize with the eisosome marker Fhn1.** Confocal images show the localization of (left panels, A-E) mGFP-tagged (A) wild-type or (B-E) mutant Myo1, (middle panels, A′-E′) mCherry-tagged Fhn1 (Fhn1-mCherry), and (right panels, A″-E″) merge of Myo1-mGFP and Fhn1-mCherry images. The wild-type and Myo1(G483D) localize to actin patches (green arrowheads) that are distinct from Fhn1-mCherry in eisosomes (red arrows). The T140I, A181P and G308R Myo1 mutants colocalize with Fhn1 in eisosomes (yellow arrows). The images represent maximum intensity projections of three consecutive optical sections through the top surface of the cell acquired at 0.4-µm intervals. Scale bar: 10 µm.

Elongated cortical structures decorated by mGFP-tagged T140I, A181P and G308R Myo1 motor domain mutants were reminiscent of eisosomes. Eisosomes are membrane invaginations/microdomains characterized by the presence of specific membrane-binding and transmembrane proteins, including Fhn1 (Kabeche et al., 2011; Moreira et al., 2009; Olivera-Couto et al., 2011; Walther et al., 2007). To determine whether Myo1 motor domain mutants indeed localized to eisosomes, we combined by genetic crosses mCherry-tagged eisosome marker Fhn1 with mGFP-tagged wild-type or mutant Myo1 (Fig. 4). Fluorescence imaging revealed colocalization of Myo1 mutants T140I, A181P and G308R with Fhn1 in eisosomes (Fig. 4C-E″, yellow arrows), whereas wild-type Myo1 or G483D mutant localized to actin patches (Fig. 4A-B″, green arrowheads), which showed little overlap with Fhn1-mCherry-labeled eisosomes (Fig. 4A-B″, red arrows). Thus, disruption of the Myo1 motor domain by kidney-disease-associated mutations resulted in targeting of Myo1 to eisosomes. The association of Myo1 mutants with eisosomes seemed to have no effect on eisosome formation because the number of eisosomes in wild-type and *myo1* mutant cells was approximately the same (supplementary material Fig. S3), despite differences in the amount of Myo1 associated with eisosomes among Myo1 mutants (supplementary material Fig. S2).

**Fig. 5.**
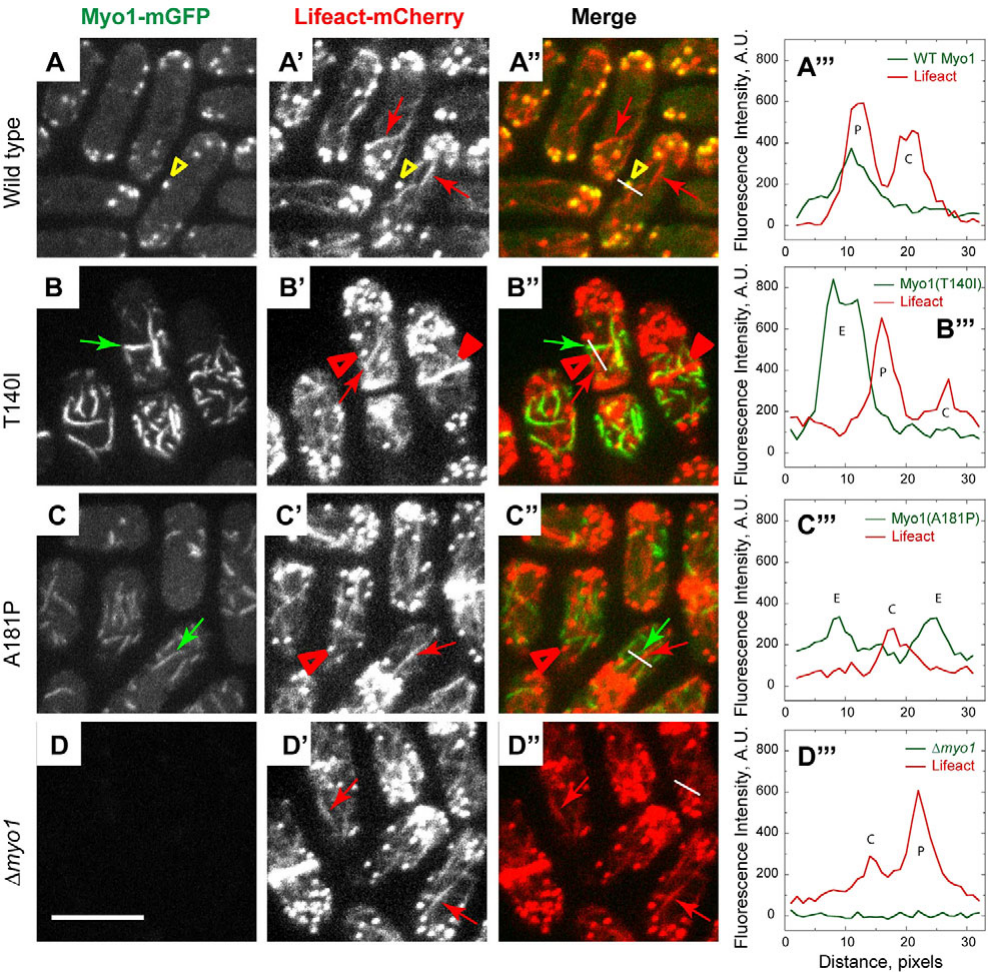
**Myo1 mutants do not colocalize with filamentous actin labeled by Lifeact-mCherry.** Confocal images show the localization of (left panels, A-D) mGFP-tagged (A) wild-type and (B-D) mutant Myo1, (panels A′-D′) Lifeact-mCherry, (panels A″-D″) merge of Myo1-mGFP and Lifeact-mCherry images, and (right panels, A‴-D‴) line scans in green (green lines) and red (red lines) channels along the white lines indicated on the merged images in panels A″-D″. Wild-type Myo1 colocalizes with Lifeact in actin patches (yellow arrowheads) but not in actin cables (red arrows). The T140I and A181P Myo1 mutants localize to eisosomes (green arrows, E in line scans) that are distinct from actin cables (red arrows, C in line scans), patches (open red arrowheads, P in line scans) and rings (filled red arrowheads in B′-B″). The images represent maximum intensity projections of three consecutive optical sections through the top surface of the cell acquired at 0.4-µm intervals. Scale bar: 10 µm.

To test whether mutant Myo1 in eisosomes can recruit any filamentous actin (F-actin) and, conversely, whether any eisosome-localizing Myo1 mutants can associate with actin patches, cables and rings, which are the three actin structures in fission yeast cells, we examined the localizations of mGFP-tagged wild-type and mutant Myo1 variants relative to the actin structures labeled with a general F-actin marker, Lifeact-mCherry (Fig. 5). In the wild-type cells, Myo1 colocalized with Lifeact in patches (Fig. 5A-A‴, yellow arrowheads) but not in cables (Fig. 5A-A‴, red arrows) or contractile actin rings. In contrast, two kidney-disease-associated mutants, Myo1(T140I)-mGFP and Myo1(A181P)-mGFP, localized to eisosomes (Fig. 5B-C‴, green arrows) that were clearly distinct from any Lifeact-labeled F-actin structures in the cells (Fig. 5B-C‴, red arrows, filled and open arrowheads). In the two mutants, as in *Δmyo1* cells, all F-actin structures, including patches, lacked any Myo1 and, conversely, no Lifeact-labeled F-actin was detected associated with Myo1 mutants in eisosomes.

### Myo1 mutants colocalize with the Cam2 light chain

Increased degradation of the A181P and G308R Myo1 mutants compared with the T140I mutant or wild-type Myo1 in immunoblotting assays (Fig. 1) suggested that their protein stability was decreased, possibly due to misfolding. To directly test whether FSGS-associated mutations affect myosin folding and stability, we examined whether the stability of Myo1 motor mutants correlated with their ability to associate with a myosin chaperone, Rng3. Rng3 is a member of the UCS family of molecular chaperones that is necessary for the motor activity of fission yeast myosin II (Myo2) (Lord and Pollard, 2004; Stark et al., 2013). Previous work showed that, although Rng3 is not required for Myo1 folding, a destabilizing G308R mutation in the Myo1 motor domain promotes recruitment of Rng3 to Myo1, which was detected using Myo1-specific light chain Cam2 tagged with mCherry (Stark et al., 2013). In *myo1(G308R)* cells, Rng3 and Cam2 colocalized in patch-like structures that, we suspect, represented cross-sections of eisosomes or protein aggregates.

**Fig. 6.**
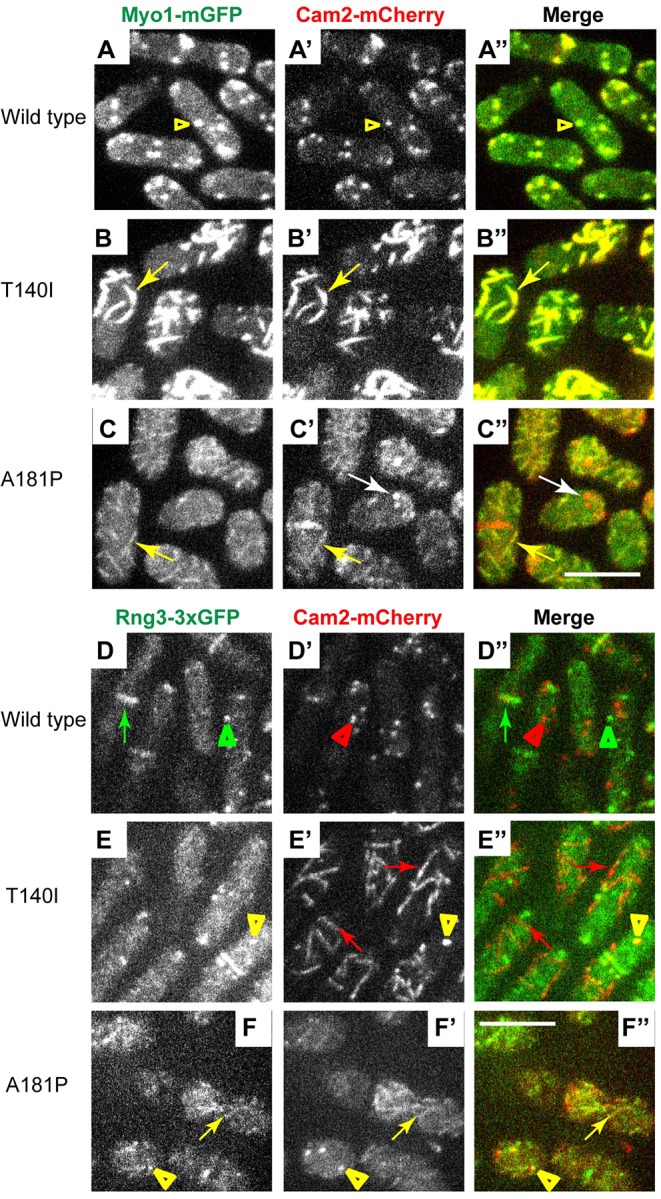
**Myo1 light chain Cam2 colocalizes with Myo1 mutants, and with Rng3 in cells expressing A181P but not the T140I Myo1 mutants.** (A-C″) Confocal images show the localization of (left panels, A-C) mGFP-tagged (A) wild-type and (B,C) mutant Myo1, (middle panels, A′-C′) mCherry-tagged Cam2 (Cam2-mCherry), and (right panels, A″-C″) merge of Myo1-mGFP and Cam2-mCherry images. Cam2-mCherry colocalizes with mGFP-tagged wild-type Myo1 in patches (yellow arrowheads) and with mGFP-tagged T140I and A181P Myo1 mutants in eisosomes (yellow arrows). In the A181P mutant, Cam2 also localizes to cytoplasmic puncta that are devoid of Myo1 (white arrows). (D-F″) Confocal images show the localization of (left panels, D-F) triple GFP-tagged Rng3 (Rng3-3×GFP), (middle panels, D′-F′) mCherry-tagged Cam2 (Cam2-mCherry), and (right panels D″-F″) merge of Rng3-3×GFP and Cam2-mCherry images in yeast strains expressing untagged wild-type or kidney-disease-associated Myo1 variants. In the wild-type cells, Rng3 localizes to the contractile rings (green arrows) and cytoplasmic spots of unknown nature (green arrowheads) that are distinct from Cam2 in actin patches (red arrowheads). Rng3 does not colocalize with Cam2 in eisosomes (red arrows in E′-E″) in the T140I mutant, but colocalizes with Cam2 in eisosomes (yellow arrows in F-F″) in the A181P mutant. Cam2 and Rng3 also colocalize in cortical puncta in A181P and, occasionally, T140I mutants (yellow arrowheads in E-F″). The images represent maximum intensity projections of three consecutive optical sections through the top surface of the cell acquired at 0.4-µm intervals. Scale bars: 10 µm.

Initially, we followed the approach taken by Stark et al. (2013) and tested whether Myo1-specific light chain Cam2 tagged with mCherry can be used to track localization of FSGS-associated Myo1 mutants in cells. We combined Cam2-mCherry with mGFP-tagged wild-type or mutant Myo1 by genetic crosses (Fig. 6A-C″, supplementary material Fig. S4A-C″) and examined their localization in live fission yeast cells. Fluorescence imaging showed that wild-type Myo1 and Myo1(G483D) colocalized with Cam2 (Fig. 6A-A″, supplementary material Fig. S4A-A″) in 100% and 91% of endocytic actin patches, respectively (supplementary material Fig. S5). Cam2 binds the second IQ motif of Myo1 (Sammons et al., 2011). When Cam2 binding to Myo1 was disrupted by a point mutation (Sammons et al., 2011) or by the deletion of the second IQ motif (supplementary material Fig. S4C-C″), Myo1 continued to localize to actin patches, whereas Cam2 re-localized to mobile and stationary puncta that were clearly distinct from actin patches (white arrows in supplementary material Fig. S4C-C″). The residual 5-12.5% colocalization between mGFP-Myo1(ΔIQ2) and Cam2-mCherry (supplementary material Fig. S5) represented occasional fortuitous juxtaposition of actin patches and Cam2 puncta. Cam2 maintained association with Myo1 motor domain mutants and Cam2-mCherry showed robust colocalization with mGFP-tagged T140I, A181P and G308R Myo1 mutants (yellow arrows in Fig. 6B-C″, supplementary material Fig. S4B-B″) in 100% of eisosomes (supplementary material Fig. S5). For each mutant, the amount of Cam2 in eisosomes correlated with the amount of Myo1 in eisosomes and the level of full-length Myo1 detected by immunoblotting (Fig. 1). In the stable T140I mutant, virtually all Cam2 colocalized with Myo1 in eisosomes, whereas, in the unstable A181P and G308R mutants, Cam2 localized to both eisosomes and cytoplasmic puncta (supplementary material Fig. S5, white arrows in Fig. 6C-C″ and supplementary material Fig. S4B-B″) similar to those observed in the cells expressing Myo1 mutant lacking the second IQ motif (supplementary material Fig. S5, white arrows in supplementary material Fig. S4C-C″). Colocalization of Cam2 with Myo1 mutants in eisosomes, structures with distinctive linear organization, allowed us to use Cam2 as a proxy for Myo1 localization.

### Chaperone Rng3 is recruited to the A181P but not the T140I Myo1 mutant

To test whether Myo1 motor domain mutants recruit Rng3, we constructed haploid yeast strains combining triple GFP-tagged Rng3 (Rng3-3×GFP) with Cam2-mCherry and the untagged versions of either the wild-type or mutant Myo1 (Fig. 6D-F″ and supplementary material Fig. S4D-F″). In the wild-type and *myo1(G483D)* cells, Rng3-3×GFP localized to actin contractile rings (green arrows in Fig. 6D-D″ and supplementary material Fig. S4D-D″) and cytoplasmic puncta (green arrowheads in Fig. 6D-D″ and supplementary material Fig. S4D-D″), and did not colocalize with Cam2-mCherry in actin patches (red arrowheads in Fig. 6D-D″ and supplementary material Fig. S4D-D″). In contrast to the wild-type cells, in *myo1(A181P)* and *myo1(G308R)* mutants, Rng3-3×GFP partially colocalized with Cam2-labeled eisosomes (yellow arrows in Fig. 6F-F″ and supplementary material Fig. S4E-E″), indicating recruitment of Rng3 to mutated Myo1. In addition to eisosomes, we also observed that, in *myo1(T140I)*, *myo1(A181P)* and *myo1(G308R)* cells, Cam2 and Rng3 colocalized in bright cortical puncta of unknown nature (yellow arrowheads in Fig. 6E-F″ and supplementary material Fig. S4E-E″), which might represent fragmented eisosomes or protein aggregates. In stark contrast to the *myo1(A181P)* and *myo1(G308R)* mutants, in *myo1(T140I)* mutant cells Rng3 was not recruited to the eisosomes (red arrows in Fig. 6E-E″), even though Cam2 in this mutant accumulated in eisosomes to a greater degree than in A181P or G308R mutants. We also found that, in the *myo1(2xIQ1IQ2)* mutant, containing normal Myo1 motor domain but duplicated IQ motifs in the neck, Cam2 localized to eisosomes (red arrows in supplementary material Fig. S4F′-F″) and did not colocalize with Rng3. Thus, eisosome localization of Myo1-bound Cam2 is not always accompanied by the Rng3 recruitment.

Although the differences in association with Rng3 among Myo1 motor domain mutants were readily apparent, the detailed analysis of colocalization was complicated by the variability in Cam2-mCherry localization to eisosomes in A181P and G308R mutants combined with Rng3-3×GFP. The fluorescence signal was weak, and some cells did not exhibit clear Cam2 enrichment in eisosomes, which could be due to the effects of combining bulky mCherry and triple GFP tags. Therefore, to directly confirm association of Rng3 with A181P and G308R but not T140I Myo1 mutants in eisosomes, we employed two additional complementary approaches. We combined wild-type and mutant Myo1 labeled with a fluorescent protein tag directly on the Myo1 heavy chain with Rng3 labeled with a contrasting fluorescent protein. Specifically, we combined mCherry-tagged Myo1 variants with Rng3-3×GFP, and mGFP-tagged Myo1 variants with Rng3-mCherry, and optimized imaging conditions to allow detection of faint mCherry-tagged proteins in live cells.

**Fig. 7.**
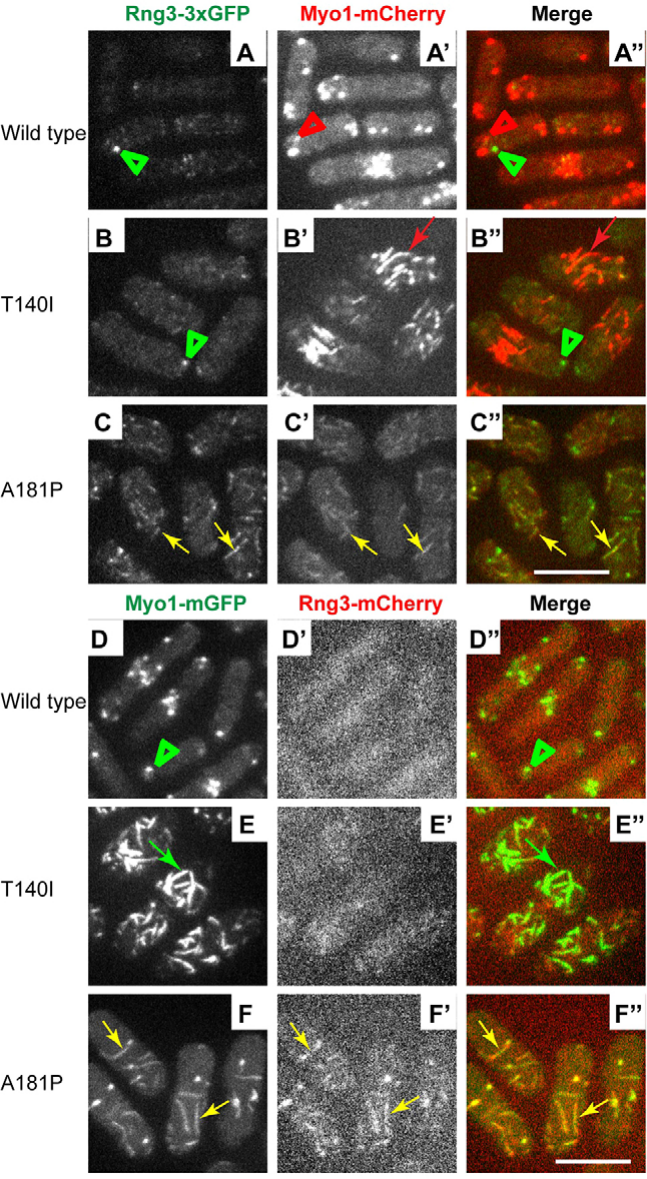
**Chaperone Rng3 is recruited to the A181P but not the T140I Myo1 mutant in eisosomes.** (A-C″) Confocal images show the localization of (left panels, A-C) triple GFP-tagged Rng3 (Rng3-3×GFP), (middle panels, A′-C′) mCherry-tagged Myo1 variants (Myo1-mCherry), and (right panels A″-C″) merge of Rng3-3×GFP and Myo1-mCherry images. Wild-type Myo1 in patches (red arrowheads) and Myo1(T140I) in eisosomes (red arrows) are distinct from Rng3 puncta (green arrowheads). Rng3 colocalizes with the A181P Myo1 mutant in eisosomes (yellow arrows). (D-F″) Confocal images show the localization of (left panels, D-F) mGFP-tagged Myo1 variants (Myo1-mGFP), (middle panels, D′-F′) mCherry-tagged Rng3 (Rng3-mCherry), and (right panels D″-F″) merge of Myo1-mGFP variants and Rng3-mCherry images. Rng3 colocalizes with Myo1(A181P) in eisosomes (yellow arrows) but not with Myo1(T140I) in eisosomes (green arrows) or wild-type Myo1 in patches (green arrowheads). The images represent maximum intensity projections of three consecutive optical sections through the top surface of the cell acquired at 0.4-µm intervals. Scale bars: 10 µm.

Fluorescence imaging revealed unequivocal association of Rng3 with A181P and G308R Myo1 mutants but not T140I Myo1 mutant. In cells combining mCherry-tagged Myo1 constructs with 3×GFP-tagged Rng3 (Fig. 7A-C″ and supplementary material Fig. S6A-B″), wild-type Myo1 and Myo1(G483D) localized to patches that lacked any Rng3 (Fig. 7A-A″ and supplementary material Fig. S6A-A″), whereas Myo1(T140I), Myo1(A181P) and Myo1(G308R) localized to eisosomes in all cells. mCherry-tagged A181P and G308R mutants in eisosomes showed robust colocalization with Rng3-3×GFP in every cell in the population (yellow arrows in Fig. 7C-C″ and supplementary material Fig. S6B-B″). In contrast, despite stronger accumulation in eisosomes, Myo1(T140I) showed little colocalization with Rng3 (Fig. 7B-B″), except a few rare examples of weak association of Rng3 with Myo1(T140) in only one or two eisosomes in less than 10% of the cells. We also noted occasional examples of Rng3 localization with T140I, A181P and G308R Myo1 mutants in cytoplasmic puncta, which we suspect are protein aggregates. Similar localization patterns were observed in a reciprocal experiment using mGFP-tagged Myo1 variants combined with mCherry-tagged Rng3 (Fig. 7D-F″, supplementary material Fig. S6C-E″). Even though the Rng3-mCherry signal was extremely faint, Rng3-mCherry showed robust colocalization in eisosomes with mGFP-tagged A181P and G308R Myo1 mutants in every cell (yellow arrows in Fig. 7F-F″ and supplementary material Fig. S6D-D″). In contrast, little or no Rng3-mCherry colocalized with mGFP-tagged T140I mutant (Fig. 7E-E″) or mutant with duplicated IQ motif in eisosomes (supplementary material Fig. S6E-E″). Rng3-mCherry also failed to colocalize with wild-type and G483D mutant Myo1 in actin patches (Fig. 7D-D″ and supplementary material Fig. S6C-C″). Thus, association of Rng3 with Myo1 is only observed with unstable Myo1 motor domain mutants.

## DISCUSSION

### FSGS-associated mutations disrupt myosin-1 function

We have examined the functional effects of FSGS-associated mutations in Myo1e by generating equivalent mutations in the fission yeast Myo1. The introduction of the T140I or A181P mutations in the *S. pombe* Myo1 motor domain led to defects in yeast growth under the high-salt and high-temperature conditions, resulting in a phenotype similar to that of the *Δmyo1* strain. Furthermore, actin patches in the Myo1 motor domain mutant strains exhibited slower patch assembly and defective patch internalization, similar to the actin patches in the *Δmyo1* cells. These observations indicate that both mutations result in the loss of myosin functional activity. Taken together with our previous findings that *Myo1e*-null mice develop FSGS-like disease, and that cultured podocytes expressing Myo1e(A159P) exhibit defects in the formation of cell-cell contacts (Bi et al., 2013; Krendel et al., 2009), our data strongly indicate that the loss of function of Myo1e is the cause of kidney disease in individuals who are homozygous for the T119I or A159P mutations (Al-Hamed et al., 2013; Mele et al., 2011; Sanna-Cherchi et al., 2011). Thus, our findings confirm that both mutations in Myo1e are likely to be pathogenic, and provide evidence that a functional Myo1e motor domain is required to maintain normal glomerular filtration.

### Molecular basis for the effects of motor domain mutations on myosin activity and stability

The two FSGS mutations examined in this study affect highly conserved residues in the myosin motor domain. The conserved Alanine at position 181 is located adjacent to the Switch I region, which is involved in coordinating actin binding with the conformational changes that occur during ATP hydrolysis (Sweeney and Houdusse, 2010). We have observed that the A181P mutant is both functionally defective and unstable, exhibiting increased degradation and association with the Rng3 chaperone. A rigid Proline residue replacing the Alanine in this position might interfere not only with the conformational changes required for motor activity but also with protein folding, thereby destabilizing the protein.

Threonine 140 is part of the P-loop consensus sequence (GXXXXGKT/S) (Smith and Rayment, 1996), also known as a Walker A motif (Walker et al., 1982), a conserved nucleotide-binding motif present in a variety of nucleotide-hydrolyzing enzymes (Koonin, 1993). The hydroxyl group of this amino acid residue forms a part of the cation-binding pocket that stabilizes the Mg^2+^ ion associated with the bound nucleotide, and, therefore, it is clearly important for ATP binding and/or hydrolysis. Indeed, mutations of this Threonine in the Walker A motif have been shown to disrupt functions of several nucleotide-hydrolyzing enzymes (Hishida et al., 1999; Shen et al., 1994; Siddique and Figurski, 2012).

Characterization of the T93I mutation in the P-loop of kinesin provides insights into the effect of this mutation on a molecular motor protein. This mutation results in strong (rigor) binding of kinesin to microtubules (Nakata and Hirokawa, 1995). In the kinesin mechanochemical cycle, the ATP-bound state corresponds to the strong microtubule-binding state, and mutations disrupting ATP hydrolysis would result in rigor binding. Thus, the T-to-I mutation in the P-loop of kinesin likely inhibits ATP hydrolysis. Unlike kinesins, ATP-bound myosins exhibit weak binding to actin filaments (Hackney, 1996). Therefore, a mutation disrupting myosin ATP hydrolysis would be expected to result in weak binding of myosin to actin, which correlates with the observed lack of localization of Myo1-T140I to actin patches. Unlike A181P, T140I mutation has no effect on Myo1 stability in yeast. Because T140I mutation disrupts the motor domain without decreasing protein stability, this mutant is particularly interesting because it illuminates the role of the Myo1 motor domain in Myo1 localization and function.

### The motor domain is necessary for myosin-1 localization

Neither the T140I, nor the A181P, mutant of Myo1 localized to actin patches in fission yeast, indicating that the functional motor domain is required for Myo1 localization to actin patches. In mammalian cells, wild-type Myo1e is found both in the cytoplasm and on the plasma membrane, with some enrichment at the cell-cell junctions and on endocytic vesicles (Bi et al., 2013; Krendel et al., 2007). Similar to the yeast Myo1 mutants that no longer localize to actin patches, Myo1e-A159P and Myo1e-T119I mutants fail to localize to cell-cell junctions and intracellular vesicles (Bi et al., 2013; J.B. and M.K., unpublished observations), and exhibit a mostly cytosolic localization. On the other hand, a Myo1e construct completely lacking the motor and neck domains (Myo1e-tail) is able to localize to the plasma membrane and endocytic vesicles (Krendel et al., 2007). However, Myo1e-tail does not completely recapitulate the normal localization of the full-length Myo1e, showing an exaggerated enrichment in the plasma membrane compared to the full-length Myo1e (Bi et al., 2013). Overall, a functional motor domain seems to be necessary for the normal intracellular localization of both human Myo1e and yeast Myo1.

In addition to the lack of colocalization between the disease-associated Myo1 mutants and actin patches, we observed an unexpected enrichment of the mutant Myo1 variants in eisosomes. We hypothesize that, when mutations in the Myo1 motor domain disrupt its ability to localize to actin patches, interactions of the Myo1 tail with the lipids, such as PIP_2_ (Kabeche et al., 2014), or proteins that are enriched in eisosomes result in targeting of Myo1 to eisosomes. The connection between the lack of motor activity and eisosomal localization of Myo1 is further substantiated by the observation that a previously characterized severe motor domain mutant of Myo1, G308R, also localized to eisosomes, whereas the mild, patch-localizing motor domain mutant G483D did not localize to eisosomes. The observed role for motor activity in determining intracellular localization of Myo1 is in agreement with the finding that Myo1 lacking the motor domain also localizes to eisosomes (R.T.C., M.L.J. and V.S., manuscript in preparation).

### Roles of the Myo1e motor domain in the kidney

The fact that both FSGS-associated mutations affect motor domain function underscores the importance of Myo1e motor activity for its roles in renal filtration. The understanding of how Myo1e motor activity contributes to the normal structure and function of the glomerular filtration barrier is still incomplete. Some of the defects caused by the motor domain mutations could be due to the mis-localization of the myosin molecule. However, it is unlikely that the motor domain function is limited to determining Myo1e localization. Indeed, a headless Myo1e construct inhibits endocytosis in a dominant-negative fashion, indicating that it is unable to functionally replace full-length Myo1e (Krendel et al., 2007).

Class I myosins might act as multifunctional linkers between the actin filaments that interact with the motor domain and the plasma membrane, and membrane-associated protein complexes that bind to the tail domain (Arif et al., 2011; Bi et al., 2013; McConnell and Tyska, 2010). Myosin motor domain activity and the ability to bind to actin are likely to be important for the processes that require coordination of membrane deformation with actin filament recruitment, including cell shape changes, endocytosis, assembly of cell adhesion complexes and regulation of membrane tension. Indeed, studies in cultured cells have demonstrated contributions of Myo1e to endocytosis, regulation of membrane tension and cell adhesion dynamics (Cheng et al., 2012; Gupta et al., 2013; Krendel et al., 2009; Nambiar et al., 2009). Furthermore, podocytes that express Myo1e(A159P) as the sole source of Myo1e exhibit defects in actin assembly at cell-cell junctions (Bi et al., 2013). In the absence of a functional motor domain, Myo1e might be unable to link the plasma membrane to the underlying actin cytoskeleton or recruit actin filaments to specific membrane subdomains, leading to defects in podocyte adhesion, endocytosis and cell shape regulation. Disruption of these processes leads to defects in glomerular filtration and FSGS pathogenesis (Faul et al., 2007; George and Holzman, 2012; Lennon et al., 2014; Soda and Ishibe, 2013).

In conclusion, we have established the fission yeast *S. pombe* as an effective model system for testing the effects of kidney-disease-associated mutations on myosin-1 function. By using this simple but powerful system, in which localization and function of myosin-1 are well defined, we have gained insights into the role of myosin-1 motor activity that are also applicable to human kidney biology.

## MATERIALS AND METHODS

### Yeast strains

Yeast strains used in this study are listed in supplementary material Table S1. All strains were constructed by genomic integrations using homologous recombination (Bähler et al., 1998; Sammons et al., 2011) and genetic crosses. The *myo1-E1* (G308R) and *myo1-S1* (G483D) mutants were generated previously (Stark et al., 2013) based on the sequence homology with *myo2-E1* and *myo2-S1* mutants (Balasubramanian et al., 1998; Wong et al., 2000).

Nucleotide changes corresponding to the T140I and A181P mutations were introduced into the *S. pombe myo1* gene sequence using the QuickChange kit (Stratagene; La Jolla, CA) according to the manufacturer's instructions. The mutations were generated in the pBS-myo1 construct that contains a 5-kb *Eco*R1 fragment of *S. pombe* genomic DNA encompassing the *myo1* gene (Lee et al., 2000). To integrate these mutations into the endogenous *myo1* locus, mutated constructs were digested with *Eco*R1, and 5-kb *myo1* gene fragments were introduced via lithium acetate transformation into the *myo1Δ::ura4+* strain (TP192), in which the *myo1* open reading frame is replaced with the ura4+ nutritional marker (Sirotkin et al., 2005), followed by counter-selection against *ura4+* on EMM plates containing 2 mg/ml 5-FOA and 0.05 mg/ml uracil. Successful integrations of mutated *myo1* sequences were confirmed by sequencing of PCR-amplified fragments of *myo1* locus.

To tag wild-type and mutant Myo1 with mGFP or mCherry at the C-terminus, the stop codon in the endogenous *myo1* gene in normal chromosomal location was replaced with gene-tagging cassettes PCR-amplified from pFA6a-mGFP-kanMX6 (pJQW 85-4) for mGFP (Bähler et al., 1998; Wu et al., 2003), or pFA6a-mCherry-natMX6 (pKS391) for mCherry (Snaith et al., 2005).

Haploid strains combining mutants with mGFP- or mCherry-tagged markers were generated by genetic crosses. Parental strains were crossed on ME plates followed by tetrad dissection directly from the cross or from the sporulating diploids isolated from the crosses. For some crosses, the G308R mutant was transformed with pUR-myo1+ plasmid, which facilitates crosses and is lost upon sporulation. During construction of the strains, no effects of tagging Myo1 mutants with fluorescent protein tags or combining these mutants with fluorescent-protein-tagged Fim1, Cam2, Lifeact or Rng3 on cell viability were observed.

### Fluorescence microscopy

Fission yeast cells were grown at 25°C in YE5S medium to exponential phase. Cells were then washed in EMM medium and mounted on pads of 25% gelatin in EMM under coverslips. The images were taken using a PerkinElmer (Waltham, MA) UltraView VoX Spinning Disc Confocal system mounted on a Nikon Eclipse Ti-E microscope equipped with Hamamatsu C9100-50 EMCCD camera and a 100×/1.4 N.A. PlanApo objective, and controlled by Volocity software. The Z-series through the entire cells were captured at 0.4-µm intervals, and the time-lapse images in a single confocal section in the middle plane of the cell were collected at 2-s intervals for 1 min. Maximum intensity projections were generated from three Z-sections through the top surface of the cells.

### Image analysis

All image analysis was performed in ImageJ (National Institutes of Health, Bethesda, MD). Brightness and contrast were adjusted identically within each figure. For actin patch tracking, at least five patches were selected from a time-lapse movie for each strain. The patch fluorescence intensity, position and movement were measured in ImageJ. Time courses of intensity and distance from origin for individual patches were aligned in time to peak intensity of Fim1-mCherry (time zero) and averaged at each time point. The whole-cell intensities in the GFP (Fig. 1G) channel were measured for five cells in frame 4 of the time series in a single confocal section through the middle of the cells and subtracted for extracellular background. Colocalization between mGFP-tagged Myo1 and mCherry-tagged Fimbrin (supplementary material Fig. S2A) was measured in ImageJ by selecting all Fim1-mCherry patches at frame 10 and counting the fraction of Fim1-mCherry patches that contained mGFP-labeled Myo1 within the 10-pixel selection area centered on an Fim1-mCherry patch at any time point during the Fim1-mCherry patch lifetime. The average numbers of eisosome per surface area (supplementary material Fig. S3) were measured by counting the numbers of eisosomes in the maximum intensity projections of three Z-sections through the top surface of three cells. Colocalization between Myo1 and Cam2 in patches, puncta and eisosomes (supplementary material Fig. S5) were measured from three Z-sections through the top surface of three cells. For patches, the presence of both mGFP and mCherry signals within a 10-pixel-wide selection area centered on a patch was counted as colocalization.

### Western blots

Fission yeast cells were grown under the same conditions that were used for live cell imaging. Cells were harvested by centrifugation and processed for immunoblotting (Wu and Pollard, 2005). Pelleted cells were re-suspended in ice-cold lysis buffer U [50 mM HEPES pH7.5, 100 mM KCl, 3 mM MgCl_2_, 1 mM EGTA, 1 mM EDTA, 0.1% Triton X-100, 1 mM DTT, 1 mM PMSF and Complete (Roche, NJ) protease inhibitor cocktail]. Total protein samples were prepared by mechanical lysis with glass beads in FastPrep-24 (MP-Bio, Santa Ana, CA), followed by addition of SDS-PAGE sample buffer, 2 min incubation at 100°C and 2 min centrifugation at 18,500 ***g*** in a microcentrifuge. Samples containing equal amounts of total protein, adjusted based on quantitation of Coomassie-stained gels, were separated on 10-20% gradient SDS-PAGE gels and transferred to Immobilon-P (EMD Millipore, Billerica, MA) membranes followed by incubation with 1:3000 dilution of primary anti-GFP antibody (Pierce, Rockford, IL) overnight at 4°C and secondary horseradish-peroxidase-conjugated antibody for 1 h at room temperature. Blots were developed using Clarity™ Western ECL substrate (Bio-Rad, Hercules, CA) and imaged using a Bio-Rad (Hercules, CA) ChemiDoc MP imager.

The total and the full-length band intensities were measured in Image Lab (Bio-Rad, Hercules, CA) and normalized to the wild type (Fig. 1D-F). The fraction of the total band intensity detected in the full-length band was also calculated (supplementary material Fig. S1A).

### Cycloheximide treatment

Cycloheximide (CHX) treatment was performed as previously described (Roseaulin et al., 2013). Briefly, fission yeast cells were grown at 25°C in YE5S medium to exponential phase. 0.1 mg/ml CHX (Amresco, Solon, Ohio) were added to cells for 24 h at 25°C, and cells were lysed and processed for western blot analysis.

## Supplementary Material

Supplementary Material
