# Supplementary material for: Effects of FSGS-associated mutations on the stability and function of myosin-1 in fission yeast
Source: Dis Model Mech. 2015 Aug 1;8(8):891–902. doi: 10.1242/dmm.020214 (PMC4527290; doi:10.1242/dmm.020214)
Supplement: Supplementary Material [file supp_8_8_891__index.html]

Supplementary Material 

# Effects of FSGS-associated mutations on the stability and function of myosin-1 in fission yeast

## DMM020214 Supplementary Material

- Supplementary Material
