# Supplementary material for: Effects of FSGS-associated mutations on the stability and function of myosin-1 in fission yeast
Source: Dis Model Mech. 2015 Aug 1;8(8):891–902. doi: 10.1242/dmm.020214 (PMC4527290; doi:10.1242/dmm.020214)
Supplement: Supplementary Material [file supp_020214_DMM020214supp.pdf]

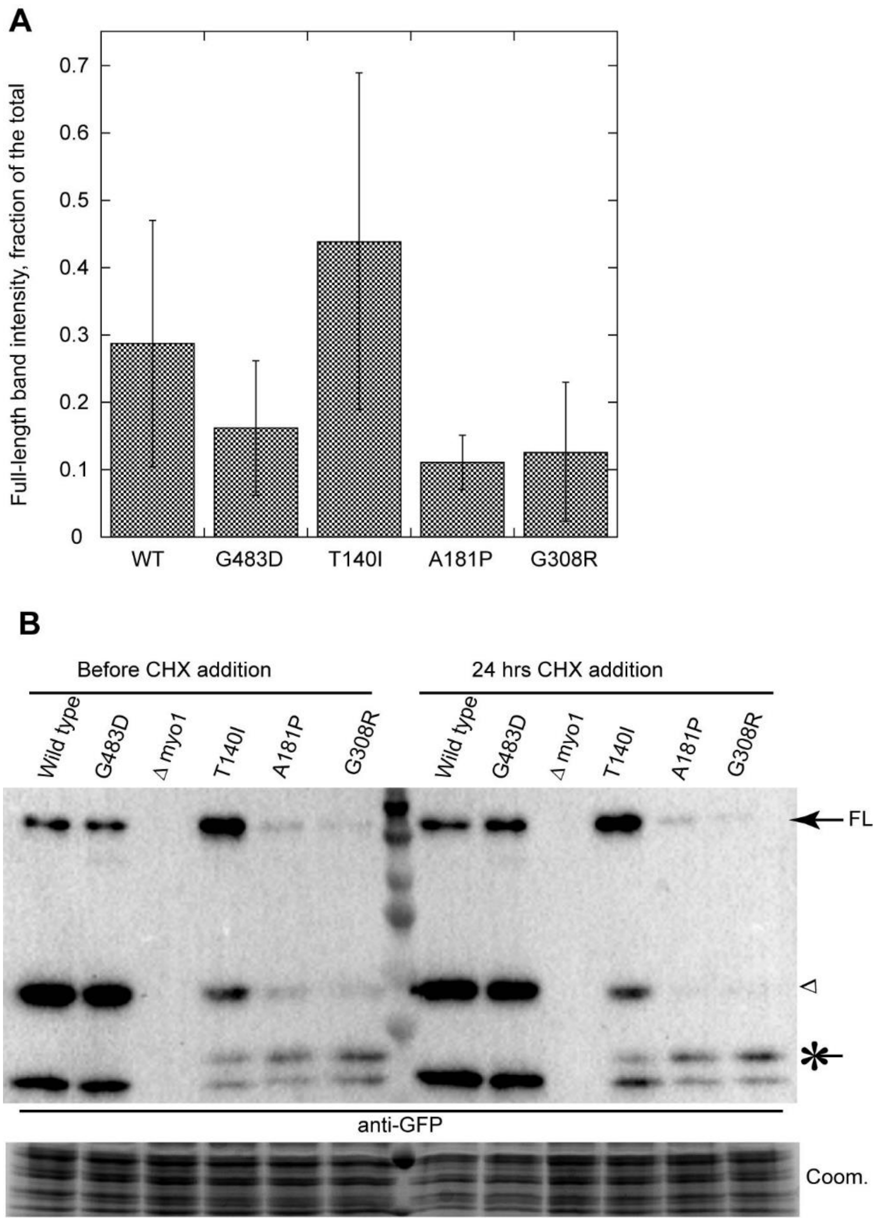

Figure S1. The A181P but not T140I mutation reduces the stability of Myo1 in cell extracts before and after cycloheximide treatment, related to Figure 1. (A) Ratio of full length band intensity to total intensity of all bands detected by Western blotting for each mGFP-tagged Myo1 variant. (B) Western blot showing mGFP-tagged Myo1 variants detected with anti-GFP antibody in extracts of cells before and after 24-hour treatment with 0.1 mg/ml cycloheximide (CHX). The arrow indicates full-length (FL) Myo1-mGFP. The arrowhead indicates a 40 kDa degradation product. The asterisk indicates a 30 kDa degradation product that is only present in T140I, A181P and G308R Myo1 mutants. Coomassie-stained gel (Coom.) serves as a loading control.

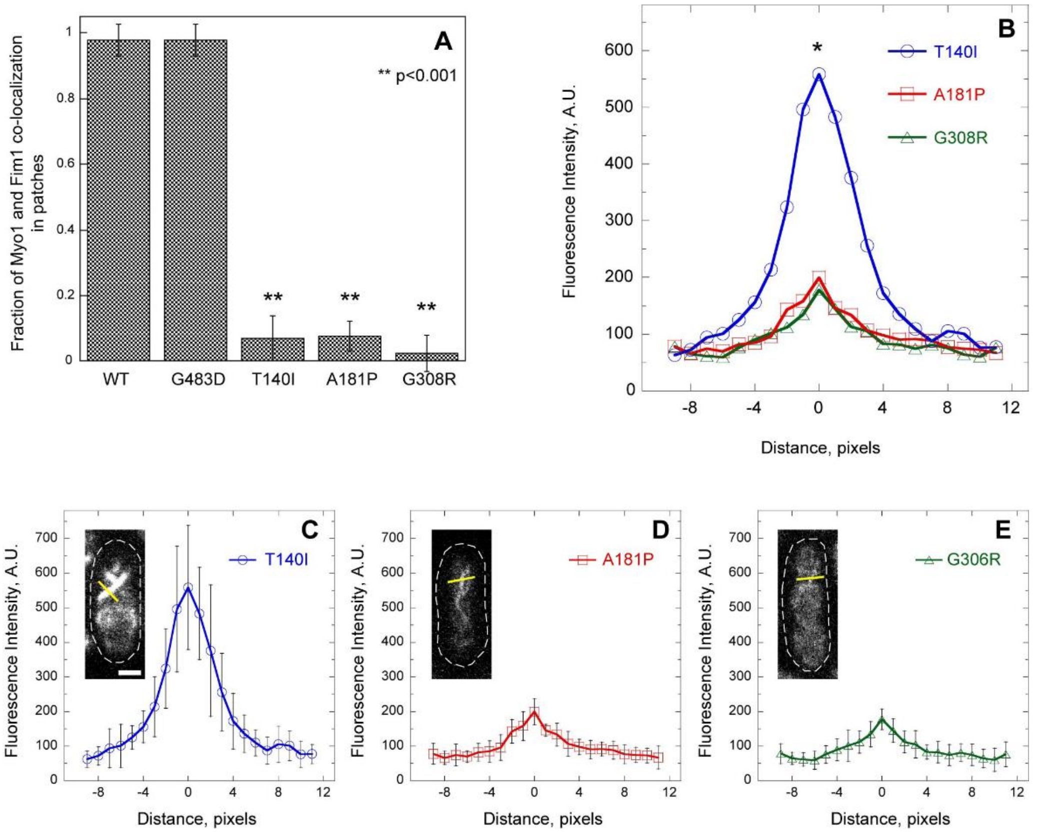

Figure S2. Motor domain mutations result in re-localization of Myo1 from actin patches to eisosomes, related to Figure 2. (A) Analysis of co-localization between mGFP-tagged Myo1 variants and Fim1-mCherry in endocytic actin patches. Fraction of Fim1-mCherry patches that contained Myo1-mGFP at any point during patch lifetime was measured in time series in single confocal sections through the middle of the cells. Error bars represent S.D. and asterisks indicate statistical significance ($p<0.001$). N= 38-55 patches in 5 cells. (B-E) Average line scans of fluorescence intensity of mGFP-tagged (blue circles) T140I, (red squares) A181P, and (green triangles) G308R Myo1 mutants in eisosomes. An asterisk indicates statistically greater ($p<10^{-5}$) peak accumulation in eisosomes for T140I mutant compared to A181P and G308R mutants. Raw line scans across 10 eisosomes for (C) T140I, (D) A181P, and (E) G308R Myo1 mutants were subtracted for extracellular background, aligned to peak intensity (zero distance), and averaged at each point. The error bars represent S.D. The insets show examples of line scans (yellow lines) across eisosomes in single confocal sections through the top surface of the cells (outlined with white dashed lines). Scale bar, 2 μm.

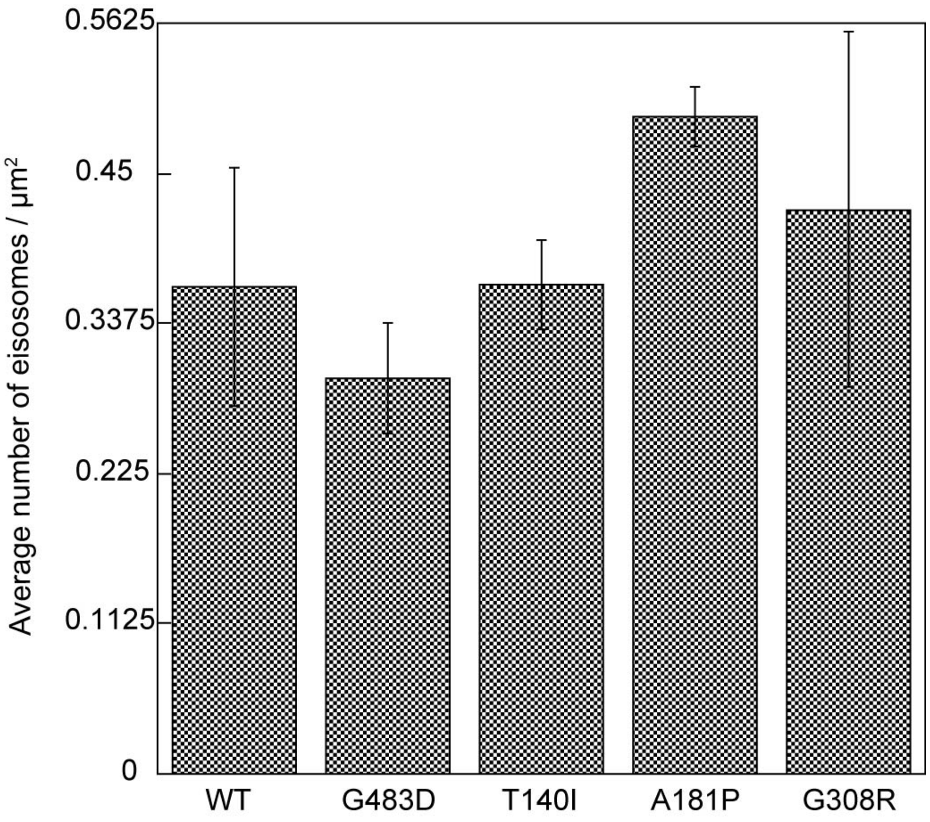

Figure S3. Recruitment of mutant Myo1 to eisosomes has no effect on the number of eisosomes in cells, related to Figure 4. The average number of Fhn1-mCherry-labeled eisosomes per µm^2 was measured from the maximum intensity projections of 3 Z-sections through the top surface of 3 randomly selected cells for each of the strains combining Fhn1-mCherry and mGFP-tagged wild type or mutant Myo1. The error bars represent S.D.

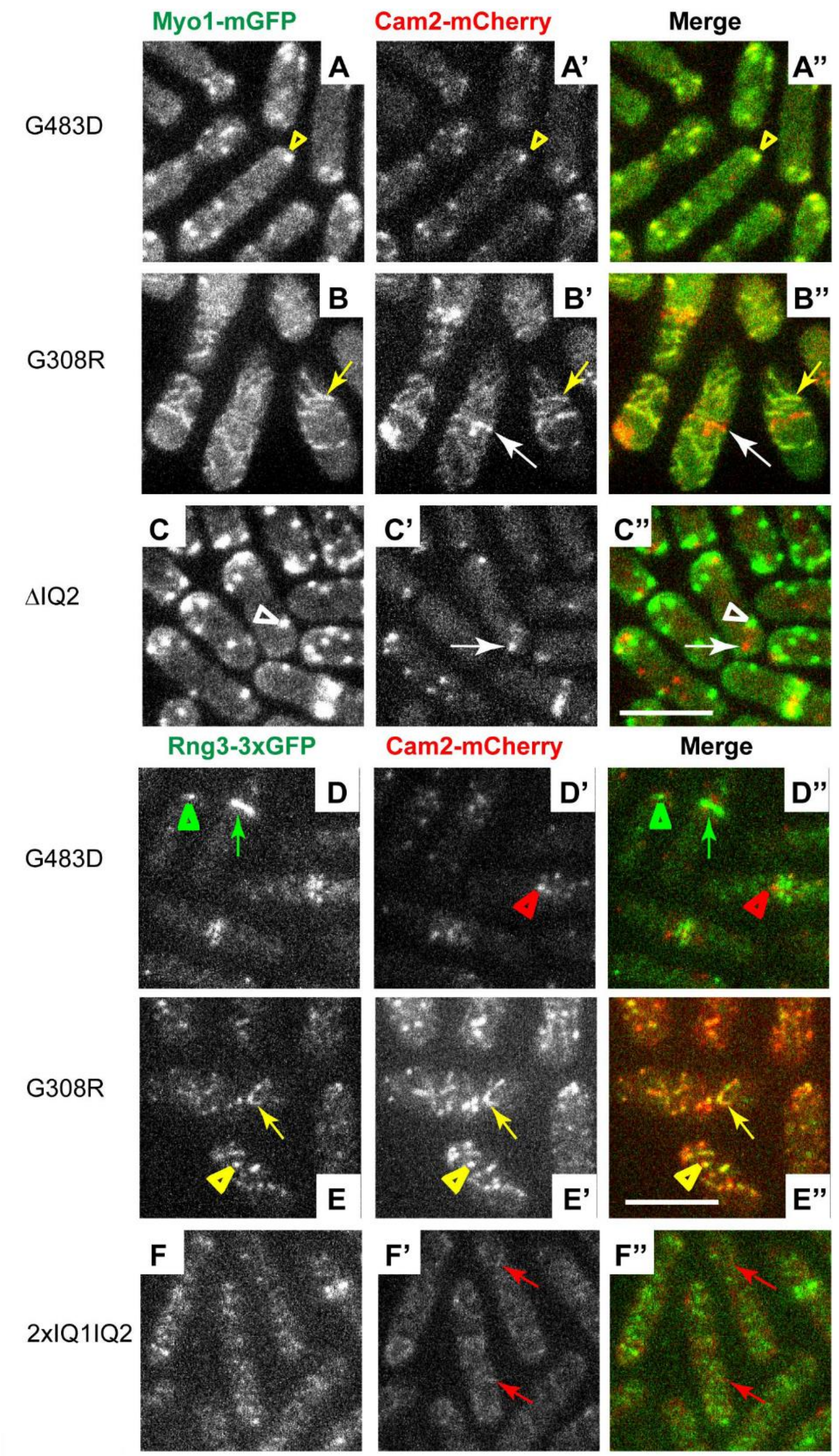

Figure S4. **Chaperon Rng3 is recruited to the G308R but not the G483D Myo1 mutant detected using Cam2-mCherry, related to Figure 6.** (A-C'') Confocal images show the localization of (left panels, A-C) mGFP-tagged mutant Myo1, (middle panels, A'-C') mCherry-tagged Cam2 (Cam2-mCherry), and (right panels, A''-C'') merge of Myo1-mGFP and Cam2-mCherry images. Cam2-mCherry co-localizes with mGFP-tagged G483D mutant Myo1 in

patches (yellow arrowheads), co-localizes with mGFP-tagged G308R Myo1 mutants in eisosomes (yellow arrows), and does not co-localize with GFP-tagged Myo1 mutant lacking the second IQ motif (ΔIQ2) in patches (white arrowheads in C-C''). In the ΔIQ2 and, occasionally, in the G308R mutant, Cam2 localizes to cytoplasmic puncta that are devoid of Myo1 (white arrows). (D-F'') Confocal images show the localization of (left panels, D-F) triple GFP-tagged Rng3 (Rng3-3xGFP), (middle panels, D'-F') mCherry-tagged Cam2 (Cam2-mCherry), and (right panels D''-F'') merge of Rng3-3xGFP and Cam2-mCherry images in yeast strains expressing untagged wild type or mutant Myo1 variants. In the G483D mutant, Rng3 localizes to the contractile rings (green arrows) and cytoplasmic spots of unknown nature (green arrowheads in D-D'') that are distinct from Cam2 in actin patches (red arrowheads). Rng3 co-localizes with Cam2 in eisosomes (yellow arrows in E-E'') in the G308R mutant and does not co-localize with Cam2 in eisosomes (red arrows in F'-F'') in the mutant with duplicated IQ motifs (2xIQ1IQ2). Cam2 and Rng3 also co-localize in cortical puncta (yellow arrowheads, E-E'') in the G308R mutant. The images represent maximum intensity projections of three consecutive optical sections through the top surface of the cell acquired at 0.4 μm intervals. Scale bar, 10 μm.

	**Myo1(+) patches**		**Myo1(+) eisosomes**		**Cam2(+) patches/puncta**		
Strain	n(N)	Fraction of Cam2(+)	n(N)	Fraction of Cam2(+)	n(N)	Fraction of Myo1(+)	Fraction of Myo1(-)
WT	3(38)	1	3(0)	NA	3(28)	1	0
G483D	3(22)	0.91	3(0)	NA	3(20)	1	0
ΔIQ2	3(20)	0.05	3(0)	NA	3(8)	0.125	0.875
T140I	3(0)	NA	3(25)	1	3(0)	0	1
A181P	3(0)	NA	3(35)	1	3(4)	0	1
G308R	3(0)	NA	3(28)	1	3(8)	0	1

n: # of cells quantified
N: # of structures
NA: Not Applicable

Figure S5. Cam2 co-localizes with Myo1 in patches and eisosomes, related to Figure 6A-C'' and Figure S4A-C''. Fractions of Myo1 patches and eisosomes that also contained Cam2 and fraction of Cam2 patches/puncta that also contained Myo1 were measured from the maximum intensity projections of 3 Z-sections through the top surface of 3 randomly selected cells for each of the strains combining Cam2-mCherry and mGFP-tagged wild type or mutant Myo1. The numbers of the structures quantified are indicated in parentheses.

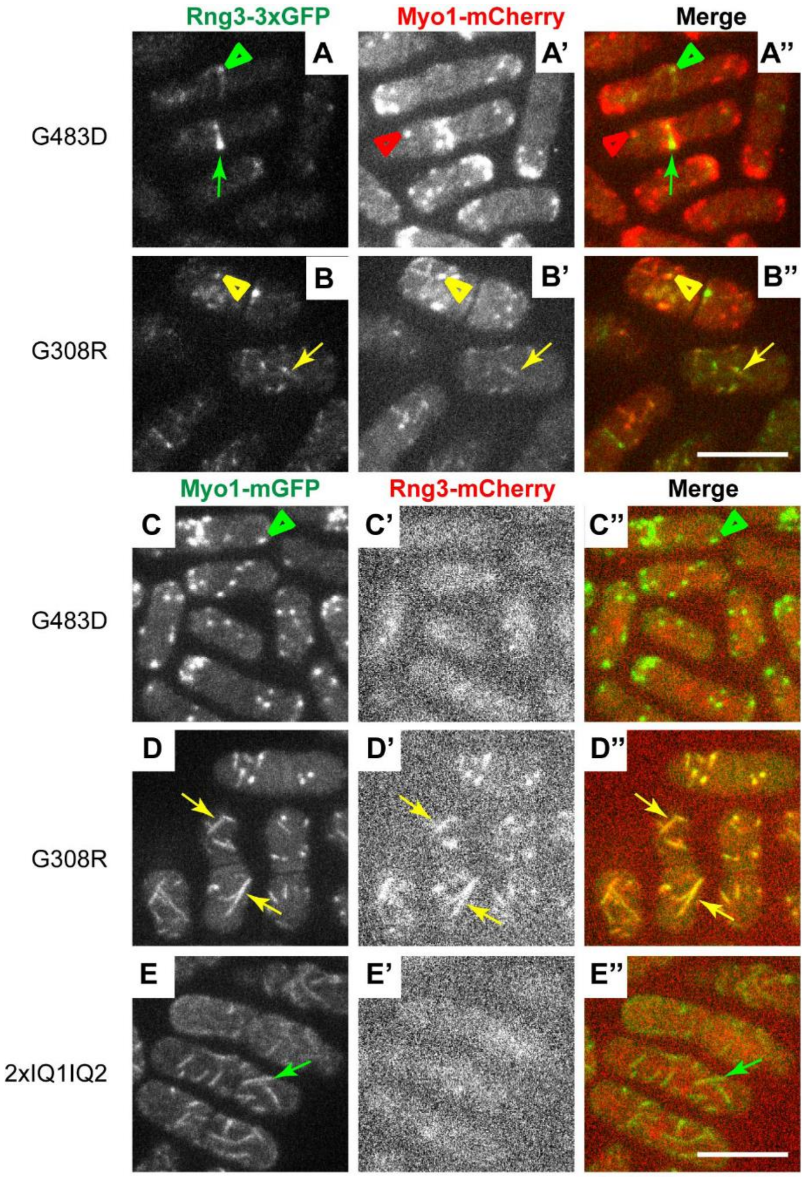

Figure S6. Chaperone Rng3 is recruited to the G308R but not the G483D Myo1 mutant, related to Figure 7. (A-B'') Confocal images show the localization of (left panels, A-B) triple GFP-tagged Rng3 (Rng3-3xGFP), (middle panels, A'-B') mCherry-tagged Myo1 variants (Myo1-mCherry), and (right panels A''-B'') merge of Rng3-3xGFP and Myo1-mCherry images. In the G483D mutant, Rng3 localizes to the contractile rings (green arrows) and cytoplasmic spots of unknown nature (green arrowheads) that are distinct from Myo1(G483D) in actin patches (red arrowheads). In the G308R mutant, Rng3 co-localizes with Myo1(G308R) in puncta (yellow arrowheads) and eisosomes (yellow arrows). (C-E'') Confocal images show the localization of (left panels, C-E) mGFP-tagged Myo1 variants (Myo1-mGFP), (middle panels, C'-E') mCherry-tagged Rng3 (Rng3-mCherry), and (right panels C''-E'') merge of Myo1-mGFP and Rng3-mCherry images. Rng3 is recruited to Myo1(G308R) in eisosomes (yellow arrows) but not to Myo1(G483D) in patches (green arrowhead) or Myo1 with duplicated IQ motifs (2xIQ1IQ2) in eisosomes (green arrow). The images represent maximum intensity projections of three consecutive optical sections through the top surface of the cell acquired at 0.4 μm interval. Scale bar, 10 μm.

Table S1. *S. pombe* strains used in this study.

Strain	Genotype	Source
	Wild type and untagged *myo1* mutants	
TP192	*h- ade6-M216 leu1-32 ura4-D18 his3-D1 Δmyo1*	Sirotkin et al. (2005)
VS685B	*h- ade6-M216 leu1-32 ura4-D18 his3-D1 myo1*	S. Forsburg
VS1139-1	*h- ade6-M216 leu1-32 ura4-D18 his3-D1 myo1(G483D)*	Stark et al. (2013)
VS1191-1	*h- ade6-M216 leu1-32 ura4-D18 his3-D1 myo1(G308R)*	Stark et al. (2013)
VS1431	*h- ade6-M216 leu1-32 ura4-D18 his3-D1 myo1(G308R) pUR-myo1$^+$::ura4$^+$*	This study
VS1828-1	*h- ade6-M216 leu1-32 ura4-D18 his3-D1 myo1(A181P)*	This study
VS1829-1	*h- ade6-M216 leu1-32 ura4-D18 his3-D1 myo1(T140I)*	This study
	mGFP-tagged Myo1 strains	
VS1254	*h- ade6-M216 leu1-32 ura4-D18 his3-D1 myo1(G483D)-mGFP-kanMX6*	Stark et al. (2013)
VS1255	*h- ade6-M216 leu1-32 ura4-D18 his3-D1 myo1(G308R)-mGFP-kanMX6*	Stark et al. (2013)
VS1256	*h+ ade6-M210 leu1-32 ura4-D18 his7-366 myo1-mGFP-kanMX6*	Stark et al. (2013)
VS1432	*h- ade6-M216 leu1-32 ura4-D18 his3-D1 myo1(308)-mGFP-kanMX6 pUR-myo1$^+$::ura4$^+$*	This study
VS1456-2	*h+ ade6-M210 leu1-32 ura4-D18 his3-D1 myo1-mGFP-kanMX6*	This study
VS1844-2	*h- ade6-M216 leu1-32 ura4-D18 his3-D1 myo1(T140I)-mGFP-kanMX6*	This study
VS1869-1	*h- ade6-M216 leu1-32 ura4-D18 his3-D1 myo1(A181P)-mGFP-KanMX6*	This study
	mCherry-tagged Myo1 strains	
VS1457C	*h+ ade6-M210 leu1-32 ura4-D18 his3-D1 myo1-mCherry-kanMX6*	This study
VS1843-1	*h- ade6-M216 leu1-32 ura4-D18 his3-D1 myo1(G483D)-mCherry-natMX6*	This study
VS1867-2	*h- ade6-M216 leu1-32 ura4-D18 his3-D1 myo1(T140I)-mCherry-natMX6*	This study
VS1868-1	*h- ade6-M216 leu1-32 ura4-D18 his3-D1 myo1(A181P)-mCherry-natMX6*	This study
VS1887-2	*h- ade6-M216 leu1-32 ura4-D18 his3-D1 myo1(G308R)-mCherry-natMX6*	This study
	Fim1-mCherry strains	
VS1263-7D	*h+ ade6-M216 leu1-32 ura4-D18 his3-D1 fim1-mCherry-natMX6 myo1Δ::kanMX6*	This study
VS1269-2A	*h+ ade6-M216 leu1-32 ura4-D18 his- fim1-mCherry-natMX6 myo1-mGFP-kanMX6*	This study
VS1270-3B	*h? ade6-M216 leu1-32 ura4-D18 his3-D1 fim1-mCherry-natMX6 myo1(G308R)-mGFP-kanMX6*	This study

VS1271-12A	*h- ade6-M216 leu1-32 ura4-D18 his3-D1 fim1-mCherry-natMX6 myo1(G483D)-mGFP-kanMX6*	This study
VS1872-3A	*h? ade6-M216 leu1-32 ura4-D18 his3-D1 fim1-mCherry-natMX6 myo1(T140I)-mGFP-kanMX6*	This study
VS1873-7A	*h? ade6-M216 leu1-32 ura4-D18 his3-D1 fim1-mCherry-natMX6 myo1(A181P)-mGFP-kanMX6*	This study
VS1890-9D	*h? ade6-M216 leu1-32 ura4-D18 his3-D1 fim1-mCherry-natMX6 myo1(A181P)-mGFP-kanMX6*	This study
VS1892-6D	*h? ade6-M210 leu1-32 ura4-D18 his3-D1 fim1-mCherry-natMX6 myo1(A181P)-mGFP-kanMX6*	This study
	Cam2-mCherry strains	
VS1274	*h- ade6-M216 leu1-32 ura4-D18 his3-D1 cam2-mCherry-kanMX6*	Sammons et al. (2011)
VS1275	*h+ ade6-M210 leu1-32 ura4-D18 his3-D1 cam2-mCherry-kanMX6*	Sammons et al. (2011)
VS1285-3A	*h? ade6-M216 leu1-32 ura4-D18 his3-D1 kanMX6-Pmyo1-mGFP-myo1ΔIQ2 cam2-mCherry-kanMX6*	This study
VS1289-1A	*h? ade6-M216 leu1-32 ura4-D18 his3-D1 myo1-mGFP-kanMX6 cam2-mCherry-kanMX6*	This study
VS1290-6A	*h? ade6-M216 leu1-32 ura4-D18 his3-D1 myo1(G483D)-mGFP-kanMX6 cam2-mCherry-kanMX6*	This study
VS1465-5C	*h? ade6-M216 leu1-32 ura4-D18 his3-D1 myo1(G308R)-mGFP-kanMX6 cam2-mCherry-kanMX6*	This study
VS1874-1B	*h? ade6-M216 leu1-32 ura4-D18 his3-D1 cam2-mCherry-kan myo1(T140I)-mGFP-kanMX6*	This study
VS1875-2B	*h? ade6-M216 leu1-32 ura4-D18 his3-D1 cam2-mCherry-kan myo1(A181P)-mGFP-kanMX6*	This study
	Fhn1-mCherry strains	
VS1718	*h- ade6+ ura4+ leu1+ his+ fhn1-mCherry-natMX6*	J. Moseley
VS1747-9B	*h+ ade6-M210 ura4+ leu1+ his+ fhn1-mCherry-natMX6*	This study
VS1747-10B	*h- ade6-M210 ura4+ leu1+ his+ fhn1-mCherry-natMX6*	This study
VS1876-4C	*h? ade6-M210 leu1-32 ura4+ his3-D1 fhn1-mCherry-Nat myo1(T140I)-mGFP-kanMX6*	This study
VS1877-3C	*h? ade6-M210 leu1+ ura4+ his3-D1 fhn1-mCherry-Nat myo1(A181P)-mGFP-kanMX6*	This study
VS1878-2D	*h? ade6-M216 leu1-32 ura4-D18 his3-D1 fhn1-mCherry-Nat myo1(483)-mGFP-kanMX6*	This study
VS1881-2B	*h? ade6-M210 leu1-32 ura4+ his+ fhn1-mCherry -Nat kanMX6-Pmyo1-mGFP-myo1*	This study
VS1883-5D	*h? ade6-M210 leu1+ ura4-D18 his+ fhn1-mCherry-Nat myo1(308)-mGFP-kanMX6*	This study
	Rng3-3xGFP strains	
VS1216-8	*h+ ade6-M210 leu1-32 ura4-D18 his3-D1 myo1(2xIQ1IQ2)*	This study
VS1348-1	*h+ ade6-M210 leu1-32 ura4-D18 his3-D1 rng3-3xGFP-MX6*	This study

VS1464-3A	*h- ade6-M216 leu1-32 Ura4-D18 his3-D1 cam2-mCherry-kanMX6 rng3-3xGFP-kanMX6*	This study
VS1464-6C	*h+ ade6-M210 leu1-32 Ura4-D18 his3-D1 cam2-mCherry-kanMX6 rng3-3xGFP-kanMX6*	This study
VS1893-2A	*h- M216 leu1-32 ura4-D18 his3-D1 myo1-mCherry-kanMX6*	This study
VS1894-4D	*h? ade6-M216 leu1-32 ura4-D18 his3-D1 myo1(T140I)-mCherry-natMX6 rng3-3xGFP-kanMX6*	This study
VS1895-12D	*h? ade6-M216 leu1-32 ura4-D18 his3-D1 myo1(A181P)-mCherry-natMX6 rng3-3xGFP-kanMX6*	This study
VS1896-2A	*h? ade6-M216 leu1-32 ura4-D18 his3-D1 myo1(G483D)-mCherry-natMX6 rng3-3xGFP-kanMX6*	This study
VS1905-10D	*h? ade6-M216 leu1-32 ura4-D18 his3-D1 myo1(G308R)-mCherry-natMX6 rng3-3xGFP-kanMX6*	This study
VS1906-9D	*h? M216 leu1-32 ura4-D18 his3-D1 myo1-mCherry-kanMX6 rng3-3xGFP-kanMX6*	This study
VS1914-5D	*h? ade6-M216 leu1-32 ura4-D18 his3-D1 myo1(T140I) cam2-mCherry-kanMX6 rng3-3xGFP-kanMX6*	This study
VS1915-8D	*h? ade6-M210 leu1-32 ura4-D18 his3-D1 myo1(A181P) cam2-mCherry-kanMX6 rng3-3xGFP-kanMX6*	This study
VS1916-4A	*h? ade6-M210 leu1-32 ura4-D18 his3-D1 myo1(2xIQ1IQ2) cam2-mCherry-kanMX6 rng3-3xGFP-kanMX6*	This study
VS1917-4B	*h? ade6-M216 leu1-32 ura4-D18 his3-D1 myo1(G483D) cam2-mCherry-kan MX6 rng3-3xGFP-kan MX6*	This study
VS1919 (ML939)	*h+ ade6-M216 leu1-32 ura4-D18 his3-D1 myo1(G308R) cam2-mCherry-kanMX6 rng3-3xGFP-kanMX6*	Stark et al. (2013)
	Rng3-mCherry strains	
VS1217-1	*h- ade6-M216 leu1-32 ura4-D18 his3-D1 kanMX6-mGFP-Pmyo1-myo1(2xIQ1IQ2)*	This study
VS1920 (ML852)	*h+ ade6-M216 leu1-32 ura4-D18 his7-366 myo2-E1 rng3-mCherry-kanR*	M. Lord
VS1960-1D	*h- ade6-M216 leu1-32 ura4-D18 his- rng3-mCherry-kanR*	This study
VS1961-6A	*h+ ade6-M210 leu1-32 ura4-D18 his- rng3-mCherry-kanR*	This study
VS1955-11D	*h? ade6-M216 leu1-32 ura4-D18 his- rng3-mCherry-kanR myo1-mGFP-kanMX6*	This study
VS1956-3B	*h? ade6-M216 leu1-32 ura4-D18 his- rng3-mCherry-kanR kanMX6-mGFP-Pmyo1-myo1(2xIQ1IQ2)*	This study
VS1957-8D	*h? ade6-M216 leu1-32 ura4-D18 his- rng3-mCherry-kanR myo1(G483D)-mGFP-kanMX6*	This study
VS1958-5D	*h? ade6-M216 leu1-32 ura4-D18 his- rng3-mCherry-kanR myo1(G308R)-mGFP-kanMX6*	This study
VS1959-10B	*h? ade6-M216 leu1-32 ura4-D18 his- rng3-mCherry-kanR myo1(A181P)-mGFP-kanMX6*	This study
VS1972-1D	*h? ade6-M216 leu1-32 ura4-D18 his- rng3-mCherry-kanR myo1(T140I)-mGFP-kanMX6*	This study

	Lifeact-mCherry strains	
VS1529 (MBY 6844)	*h- ade6-M216 leu1-32 ura4-D18 leu1::Pact1-Lifeact-mCherry-leu1+*	Huang et al. (2012)
VS1669-3B	*h+ ade6-M210 ura4-D18 leu1-32::Pact1-Lifeact-mCherry-leu1+*	This study
VS1947-4D	*h? ade6-M210 ura4-D18 myo1(T140I)-mGFP-kanMX6 leu::Pact1-Lifeact-mCherry-leu+*	This study
VS1948-5C	*h? ade6-M210 ura4-D18 myo1(A181P)-mGFP-kanMX6 leu::Pact1-Lifeact-mCherry-leu+*	This study
VS1953-5A	*h? ade6-M216 ura4-D18 his3-D1 Myo1-mGFP-kanMX6 leu::Pact1-Lifeact-mCherry-leu+*	This study
VS1954-4A	*h? ade6-M21? ura4-D18 his3-D1 myo1Δ::kanMX6 leu1-32::Pact1-Lifeact-mCherry-leu+*	This study

References

Huang, J., Huang, Y., Yu, H., Subramanian, D., Padmanabhan, A., Thadani, R., Tao, Y., Tang, X., Wedlich-Soldner, R., Balasubramanian, M.K. (2012). *J Cell Biol*. **199(5)**: 831-47. doi: 10.1083/jcb.201209044.

Sammons, M. R., James, M. L., Clayton, J. E., Sladewski, T. E., Sirotkin, V. and Lord, M. (2011). A calmodulin-related light chain from fission yeast that functions with myosin-I and PI 4-kinase. *J Cell Sci* **124**, 2466-77.

Sirotkin, V., Beltzner, C. C., Marchand, J. B. and Pollard, T. D. (2005). Interactions of WASp, myosin-I, and verprolin with Arp2/3 complex during actin patch assembly in fission yeast. *J Cell Biol* **170**, 637-48.

Stark, B. C., James, M. L., Pollard, L. W., Sirotkin, V. and Lord, M. (2013). UCS protein Rng3p is essential for myosin-II motor activity during cytokinesis in fission yeast. *PLoS One* **8**, e79593.
